# Intercropping *Amomum villosum* enhances soil stratification, nutrient complementarity, and microbial communities in rubber plantations

**DOI:** 10.3389/fmicb.2025.1720828

**Published:** 2026-01-07

**Authors:** Huabo Du, Yuchen Lin, Meijun Qi, Peng Qu, Zhenhuai Xu, Rong Lin, Chun Xie, Tengwei Xiao, Shilang Dong, Butian Wang, Yu Ge

**Affiliations:** 1 College of Tropical Crops, Yunnan Agricultural University, Pu’er, Yunnan, China; 2 Institute of Plant Resources, Yunnan University, Kunming, China

**Keywords:** *Amomum villosum*, Soil stratification, *Hevea brasiliensis*, rhizosphere microbiome, soil physicochemical properties

## Abstract

Intercropping is widely promoted to sustain soil function, yet evidence for its application in rubber-based agroforestry, particularly with the shade-tolerant herb *Amomum villosum*, is limited. We evaluated whether *A. villosum* intercropping improves soil properties and reorganizes microbiomes across the vertical profile of mature rubber plantations. Soil samples were taken at 0–10, 10–20, and 20–30 cm depths in both intercropped and monoculture stands. Physical and chemical properties were quantified, and bacterial (16S rRNA V3–V4) and fungal (ITS2) communities were analyzed using high-throughput amplicon sequencing with depth-resolved data on diversity, composition, and functional inference (FAPROTAX, FUNGuild). Intercropping consistently improved soil structure and fertility, with the strongest effects at 0–10 cm. Total porosity (12%), organic matter (38.9%), alkali-hydrolyzable nitrogen (75.4%), and available phosphorus (131%) were markedly higher than in monoculture. Benefits extended to mid-depth with a 65.2% increase in alkali-hydrolyzable nitrogen. Microbial richness (bacteria and fungi) increased, and communities separated clearly by treatment and depth. Intercropped soils showed higher relative abundances of copiotrophic and particle-attached phyla (e.g., Proteobacteria, Planctomycetota), while Acidobacteriota and several Chloroflexi declined. Nitrospirota increased with depth. Fungal trophic structure shifted away from pathotrophs at 20–30 cm and toward symbiotrophs, particularly arbuscular mycorrhizal lineages, at subsurface layers. Functional predictions indicated greater potential for nitrogen transformations (e.g., nitrogen fixation, nitrification), greater C₁/hydrocarbon utilization, and a reduced bacterial plant-pathogen signal under intercropping. Collectively, *A. villosum* intercropping reorganizes the soil environment and microbiome in mutually reinforcing ways—improving physical structure, enlarging near-term nitrogen supply, and favoring beneficial fungal guilds. These depth-resolved effects help explain the agronomic appeal of rubber–*A. villosum* systems and support their wider deployment in rubber plantations.

## 1 Introduction

The rubber tree (*Hevea brasiliensis*) is a key component of tropical ecosystems, as natural rubber remains an essential industrial raw material and a strategically important commodity (Tang et al., 2016). However, the rapid expansion of rubber monocultures across Southeast Asia and Southwest China has exposed ecological and agronomic vulnerabilities. Long-term single planting often acidifies highly weathered tropical soils, reduces soil organic matter, unbalances nutrient stocks, and weakens nutrient retention (Li et al., 2007; Liu et al., 2019; Snoeck et al., 2013). At the stand and landscape scales, simplified canopy structure and understorey use can diminish biodiversity, hydrological regulation, and soil conservation. Economically, valuable inter-row space remains underutilized in monoculture systems.

Intercropping—growing two or more complementary species simultaneously—can improve the capture and partitioning of light, water, and nutrients, thereby stabilizing yields and enhancing system resilience (Zhang et al., 2007; Yu et al., 2015; Li et al., 2021; Xu et al., 2020; Pierre et al., 2022; Xiao X. et al., 2023). Belowground, diverse root architecture and litter inputs can stabilize soil aggregates, raise porosity, regulate the distribution of water with depth, and promote nutrient cycling and microbial processes (Li et al., 2016; Nyawade et al., 2019; Curtright and Tiemann, 2021; Hu et al., 2022). Importantly, agroforestry and intercropping systems also alter soil microbial communities by modifying rhizodeposition, nutrient availability, and microclimatic conditions (Lian et al., 2019; Sun et al., 2022; Shu et al., 2024). Mixed-species root systems can increase microbial biomass, enhance alpha and beta diversity, and promote beneficial taxa involved in decomposition, nutrient mineralization, and symbiosis (Cuartero et al., 2022; Zhao et al., 2022; Gao and Xie, 2023; Xiao X. et al., 2023; Tong et al., 2024). Previous agroforestry studies show that increased litter heterogeneity and complementary root exudates can restructure microbial networks, strengthen nutrient-cycling pathways, and create clearer vertical stratification of microbial assemblages compared with monocultures (Nyawade et al., 2019; Tang et al., 2022; Peng et al., 2023; Lu et al., 2025). In rubber-based systems, agroforestry has been linked to higher soil enzyme activities, reduced land-use intensity, and improved water and sediment regulation compared to monoculture (Wen et al., 2022; Xiao L. et al., 2023). At the farm scale, forest-based economies can diversify income while sustaining ecological functions, aligning with greener and more circular development pathways (Luedeling et al., 2016; de Blécourt et al., 2014; Wigboldus et al., 2017).

*Amomum villosum* Lour. is a shade-tolerant, perennial medicinal herb (Zingiberaceae) traditionally used for gastrointestinal and obstetric purposes (Wu et al., 2004; Ao et al., 2019). Its preference for humid, shaded environments makes it an ideal candidate for under-rubber intercropping. Prior work in rubber agroforestry shows that intercropping can increase soil pH, available nitrogen, total phosphorus, and organic matter (Luo et al., 2016; Li et al., 2018; Tang et al., 2022). Species such as *Acacia chinensis* and *Pandanus amaryllifolius* have improved soil organic carbon, available phosphorus, total potassium, and alkali-hydrolyzable nitrogen under rubber (Qi et al., 2024; Zhang et al., 2023).

Despite increasing interest in rubber agroforestry, rigorous evaluations of how *A. villosum* intercropping affects soil physical and chemical properties, as well as the structure and function of soil microbiomes, remain limited, especially with depth-resolved analyses that directly link changes in soil conditions to microbial assembly processes and functional potentials. Because intercropping modifies root distribution, nutrient dynamics, and resource heterogeneity with depth, we expect these shifts to generate corresponding changes in soil microbial organization. On this basis, we hypothesized that *A. villosum* intercropping would improve soil structure and nutrient availability, particularly within surface horizons, and would increase microbial richness while shifting community composition toward copiotrophic bacteria and symbiotrophic fungi with clearer depth-dependent patterns. We further anticipated that intercropping would enhance microbial functional potentials related to nitrogen, one-carbon, and sulfur cycling while reducing pathogen-associated signals. Finally, we expected that these responses would manifest as depth–treatment interactions arising from intercropping-induced modifications to soil conditions and complementary resource use by rubber and *A. villosum*.

Following were the objectives of present study: (1) Quantify soil moisture, porosity, pH, soil organic matter, total nitrogen, total phosphorus, total potassium, available phosphorus, and alkali-hydrolyzable nitrogen in intercropped versus monoculture rubber stands across 0–10, 10–20, and 20–30 cm. (2) Profile bacterial (16S rRNA V3–V4) and fungal (ITS2) communities by depth; assess alpha and beta diversity across treatments. (3) Resolve treatment- and depth-associated taxa and biomarkers shaping community composition. (4) Infer bacterial functions (FAPROTAX) and fungal trophic modes/guilds (FUNGuild) in a depth-resolved manner. (5) Link edaphic variables to microbial diversity, composition, and predicted functions to identify key drivers.

## 2 Materials and methods

### 2.1 Study site and stand description

Fieldwork was conducted in May 2024 at the Yunnan Agricultural University experimental base, located in Pogao Village, Puwen Town, Xishuangbanna Prefecture, Yunnan, China (22°27′17.4″N, 101°03′51.9″E; 858 m a.s.l.). The site has a humid tropical monsoon climate, with a mean annual temperature of 20.2 °C and annual precipitation of 1,675.6 mm.

The rubber trees (*Hevea brasiliensis* (Willd. ex A. Juss.) Müll. Arg.) variety ‘Yunyan 77–2 were planted in 2001 at 8 m row spacing × 2 m tree spacing. In April 2019, *Amomum villosum* was interplanted at 1 m × 1 m within the inter-row space, 1 m offset from the rubber rows (Supplementary Figure S1). Monoculture rubber stands (no *A. villosum*) served as control (CK). The intercropped and monoculture stands received the same irrigation schedule and fertilizer regime. Water and fertilizer management practices were kept consistent across intercropped and monoculture plots.

### 2.2 Plot layout, tree selection, and sampling design

Three representative plots were selected for each system (intercropping and monoculture). Each plot consisted of three rows, with 10 rubber trees per row. Before soil sampling, a per-tree survey was conducted to record diameter at breast height (DBH) measured at 1.30 m above ground on the upslope side and total height. At the time of the survey, trees averaged 21.6 m in height and 24.2 cm in DBH.

Around each sample tree, we established a sampling point located at half the inter-row distance (4 m) and perpendicular to the rubber row. Soil and fine roots were collected in September 2024 using an excavation monolith of 30 cm × 30 cm (area) per depth. Stratified sampling was performed at depths of 0–10 cm, 10–20 cm, and 20–30 cm to capture (i) the biologically active surface horizon influenced by litter/exudates (0–10 cm), (ii) the mid-rooting zone where *A. villosum* and fine rubber roots overlap (10–20 cm), and (iii) the upper subsoil, where water redistribution and nitrification often intensify (20–30 cm). These depth bands are standard for resolving vertical gradients in edaphic traits and microbiomes in tropical plantations (Lu, 1999; Bao, 2000). Each sample area measured 30 cm × 30 cm. Soil moisture and porosity were determined on-site using a ring knife.

For physical analyses (moisture, porosity), undisturbed cores were taken in triplicate per sampling point using a stainless ring-core (ring knife). For chemical and microbiome analyses, soils were composited by depth from three subsamples (within 0.5–1 m of the core) per plot and homogenized. Soils for chemistry were placed in zip-sealed bags, transported cooled, air-dried at room temperature, gently disaggregated, and sieved to 2 mm. Soils for microbiology were placed in sterile 50 mL tubes, immediately flash-frozen in liquid N₂, and stored at −80 °C. Roots (rubber, *A. villosum*) were collected from the profile face, placed on ice, and stored at −80 °C for later processing. The numbers and labels of root and soil samples are shown in Table 1.

**Table 1:** Sampling scheme and codes by cropping system and depth.

Cropping system	Soil depth (cm)	Sample code
Intercropped rubber-*Amomum villosum*	0–10	AV1
	10–20	AV2
	20–30	AV3
Monoculture rubber	0–10	CK1
	10–20	CK2
	20–30	CK3

AV1, AV2, AV3 = soil sampled at 0–10, 10–20, 20–30 cm soil depth, respectively, for rubber-*A. villosum* intercropping system; CK1, CK2, CK3 = soil sampled at 0–10, 10–20, 20–30 cm soil depth, respectively, for monoculture rubber.

### 2.3 Soil physical and chemical analyses

Soil moisture content (MC) was measured gravimetrically after oven drying at 105 °C. pH was determined by the water extraction (soil: water ratio 1:2.5). Soil total porosity (STP) was calculated from undisturbed ring-core bulk density using the formula (Lu, 1999; Bao, 2000):


STP=1−(BD/PD)

Where BD is the soil bulk density, and PD is the particle density (2.65 g cm^−3^).

Soil organic carbon (SOC) was determined by dischromate (K_2_Cr_2_O_7_) oxidation with external heating (Walkley-Black method; Nelson and Sommers, 1996; Lu, 1999). Soil organic matter (SOM) was calculated as SOC × 1.724. Total nitrogen (TN) was determined using an automatic Kjeldahl nitrogen analyzer (Hanon K1160, Hanon Instruments, Weifang, China). Total phosphorus (TP) and total potassium (TK) were quantified after sodium hydroxide fusion and colorimetry (molybdenum-antimony) for TP or flame photometry for TK (Lu, 1999; Bao, 2000). Alkali-hydrolyzable nitrogen (AN) was determined by the alkali-hydrolysis diffusion method (Keeney and Neslon, 1982; Lu, 1999). Available phosphorus (AP) was extracted by HCl-H_2_SO_4_ (double acid) and measured colorimetrically. Available potassium (AK) was determined by flame photometry after neutral ammonium acetate extraction.

Stoichiometric ratios of carbon-nitrogen (C/N) [Equation (1)], carbon-phosphorus ratio (C/P) [Equation (2)], and nitrogen-phosphorus (N/P) [Equation (3)] were calculated as follows:


$$C/N=\frac{\mathrm{SOC}}{\mathrm{TN}}$$ (1)

$$C/P=\frac{\mathrm{SOC}}{\mathrm{TP}}$$ (2)

$$N/P=\frac{\mathrm{TN}}{\mathrm{TP}}$$ (3)

Where SOC, TN, and TP are expressed in g/kg.

### 2.4 High-throughput sequencing of soil microorganisms

Microbial DNA was extracted from soil samples (0.5 g) using the E.Z.N.A Soil DNA Kit (Omega Bio-Tek, Norcross, GA, USA). DNA concentration and purity were assessed with a NanoDrop 2000 (Thermo Fisher Scientific, USA), and integrity was verified by 1% agarose gel electrophoresis.

The V3-V4 region of the bacterial 16S rDNA gene was amplified using primers 341F/805R, and the ITS2 region of the fungal rDNA gene with primers ITSFI2/ITS2R. The targeted 16S rRNA V3–V4 and ITS2 regions are widely adopted for profiling bacterial and fungal communities, respectively, because they balance phylogenetic resolution with Illumina read performance and enable reliable taxonomic assignment (Klindworth et al., 2013; Schoch et al., 2012).

PCR amplification was performed in a 20 μL reaction using TransStart FastPfu DNA polymerase. The thermal cycling was: 95 °C for 5 min; 30 cycles of 95 °C for 30 s, 54 °C for 30 s, and 72 °C for 45 s; followed by final extension at 72 °C for 10 min. PCR products were verified by electrophoresis, purified by gel extraction, quantified, and pooled in equimolar concentrations to construct sequencing libraries. Libraries were quality-checked and sequenced on the Illumina NovaSeq 2,500 platform (Illumina, San Diego, CA, USA). Raw reads were processed by merging overlapping sequences, filtering out low-quality sequences, and removing chimeric sequences. The raw FASTQ files corresponding to the ITS and 16S rRNA gene sequencing datasets have been deposited in the NCBI Sequence Read Archive (SRA) database. Denoising and Amplicon Sequence Variants (ASV) inference were performed using QIIME2 (DADA2). Taxonomy was assigned against SILVA v138.1 (bacteria) and UNITE v8.3 (fungi). Libraries were normalized by rarefaction to the minimum sequencing depth per dataset (rarefaction curves in Supplementary Figure S2). Good’s coverage approached 1.0 for all libraries (Supplementary Table S11), indicating adequate depth.

### 2.5 Diversity, composition, functional inference, and statistics

Alpha diversity (Observed, ACE, Shannon, Simpson, Pielou) was computed on rarefied ASV tables. Beta diversity was measured using Unweighted-UniFrac (bacteria) and Bray–Curtis (fungi); ordinations were visualized via PCoA. Soil properties were analyzed using a two-way analysis of variance (ANOVA) with fixed factors: cropping (intercropped vs. monoculture) and depth (0–10, 10–20, 20–30 cm), including the interaction. Normality and variance assumptions were checked. Post-hoc comparisons used Tukey’s HSD (*p* < 0.05). Community differences were tested by PERMANOVA (adonis2, 9,999 permutations) on the corresponding distance matrices, evaluating Cropping, Depth, and their interaction (*R*^2^ and *p* reported).

To identify depth- and treatment-associated taxa, we used LEfSe (Kruskal–Wallis + Wilcoxon; default *α* = 0.05; LDA thresholds: phyla >2.0, genera >4.0) to highlight consistent biomarkers across groups. Given multiple testing, *p*-values in univariate comparisons were FDR-adjusted (Benjamini–Hochberg). Spearman correlations linked dominant phyla to soil variables; significance was FDR-adjusted and mapped in heatmaps.

Bacterial functional potentials were inferred using FAPROTAX; fungal trophic modes/guilds were annotated with FUNGuild. We report functions/guilds as putative potentials rather than realized process rates. Analyses were conducted in R (v2025.05.0 + 496; packages: vegan, phyloseq, microeco, ggplot2, circlize, ggalluvial) and QIIME2 (2023.x). LEfSe was run in the Galaxy implementation.

## 3 Results

### 3.1 Effect of intercropping on soil properties

#### 3.1.1 Physico-chemical properties

Across both systems, soil moisture increased with depth, and soil porosity of the surface layer (0–10 cm) was higher than in deeper layers (Table 2). Intercropping rubber with *Amomum villosum* increased moisture at 0–10, 10–20, and 20–30 cm by 3.6, 0.1 and 4.4% respectively, compared to monoculture. Porosity increased by 11.6, 5.7, and 7.4% compared to monoculture, respectively. These changes indicate improved water retention and a more open pore network in intercropped soils, conditions known to favor root exploration and microbial activity.

**Table 2:** Soil physico-chemical properties by cropping system and depth (mean ± SD, *n* = 3).

Treatments	Moisture content (%)	Porosity (%)	pH	SOM (g/kg)
AV1	21.20 ± 0.01^d^	41.00 ± 0.00^a^	5.22 ± 0.00^a^	23.53 ± 0.09^a^
AV2	23.00 ± 0.00^b^	37.10 ± 0.00^b^	5.20 ± 0.00^a^	17.01 ± 0.03^b^
AV3	23.97 ± 0.01^a^	36.48 ± 0.00^d^	5.14 ± 0.00^b^	16.64 ± 0.03^d^
CK1	20.46 ± 0.00^e^	36.73 ± 0.01^c^	4.93 ± 0.00c	16.94 ± 0.13^bc^
CK2	22.98 ± 0.00^bc^	35.10 ± 0.01^e^	4.89 ± 0.00^d^	16.75 ± 0.06^cd^
CK3	22.96 ± 0.01^c^	33.96 ± 0.01^f^	4.76 ± 0.00^e^	12.98 ± 0.04^e^

Values are mean ± standard deviation (*n* = 3). Significant interaction between cropping system and soil depth was detected by two-way ANOVA (*p* < 0.05). Tukey’s honestly significant difference (HSD) post-hoc test was applied for pairwise comparisons. Within a column, different lowercase letters indicate significant differences among the six treatment combinations at *p* < 0.05. SOM: soil organic matter; AV1, AV2, AV3 = soil sampled at 0–10, 10–20, 20–30 cm soil depth, respectively, for rubber-*A. villosum* intercropping system; CK1, CK2, CK3 = soil sampled at 0–10, 10–20, 20–30 cm soil depth, respectively, for monoculture rubber.

Both pH and soil organic matter (SOM) decreased with depth in each system. Intercropping increased pH by 5.9, 6.3, and 8.0% at 0–10, 10–20, and 20–30 cm, respectively, indicating less acidic conditions (pH 5.14–5.22) rather than a shift toward alkalinity. SOM was consistently higher under intercropping, with 39, 1.6, and 28.2% increases at 0–10, 10–20, and 20–30 cm, respectively. These patterns suggest increased carbon inputs and stabilization in the topsoil, along with improved conditions at depth.

#### 3.1.2 Nutrient contents

Total nitrogen (TN) and alkali-hydrolyzable nitrogen (AN) decrease with soil depth but increase under intercropping (Table 3). TN increased by 5–28% across depths. AN showed a more substantial increase of 75.4, 65.2, and 9.4%, at 0–10, 10–20, and 20–30 cm, respectively. Total P (TP) and available P (AP) also increased under intercropping. TP increased by 15.7, 14.8, and 6%, while AP rose by 131, 72, and 14% at 0–10, 10–20, and 20–30 cm, respectively. Total K (TK) increased by more than 30% at all depths under intercropping. Available K (AK) also increased in the upper two layers (13.6% at 0–10 cm; 7.3% at 10–20 cm) but declined in the deepest layer (−14.5% at 20–30 cm), reflecting depth-dependent responses.

**Table 3:** Soil nutrients by cropping system and depth (mean ± SD, *n* = 3).

Treatments	TN (g/kg)	AN (mg/kg)	TP (g/kg)	AP (mg/kg)	TK (g/kg)	AK (mg/kg)
AV1	1.09 ± 0.01^a^	174.42 ± 2.14^a^	1.03 ± 0.01^a^	8.62 ± 0.20^a^	10.00 ± 0.27^a^	66.42 ± 3.85^a^
AV2	0.83 ± 0.01^b^	130.29 ± 0.50^b^	1.01 ± 0.02^b^	6.06 ± 0.20^b^	9.55 ± 0.06^a^	57.32 ± 2.40^c^
AV3	0.76 ± 0.02^d^	79.57 ± 0.40^d^	0.90 ± 0.01^c^	4.02 ± 0.03^c^	7.34 ± 0.27^b^	42.28 ± 3.52^d^
CK1	0.85 ± 0.01^b^	99.44 ± 2.35^c^	0.89 ± 0.01^c^	3.73 ± 0.03^d^	7.65 ± 0.06^b^	58.46 ± 1.86^b^
CK2	0.79 ± 0.02^c^	78.87 ± 1.22^d^	0.88 ± 0.01^c^	3.53 ± 0.03^d^	6.07 ± 0.66^c^	53.40 ± 0.88^bc^
CK3	0.72 ± 0.01^e^	72.72 ± 0.06^e^	0.85 ± 0.01^d^	3.53 ± 0.08^d^	5.44 ± 0.06^c^	49.48 ± 0.43^cd^

Values are mean ± standard deviation (*n* = 3). Significant interaction between cropping system and soil depth was detected by two-way ANOVA (*P* < 0.05). Tukey’s honestly significant difference (HSD) *post-hoc* test was applied for pairwise comparisons. Within a column, different lowercase letters indicate significant differences among the six treatment combinations at *p* < 0.05. TN: total nitrogen; AN: alkali-hydrolyzable nitrogen; TP: total phosphorus; AP: available phosphorus; TK: total potassium; AK: available potassium. AV1, AV2, AV3 = soil sampled at 0–10, 10–20, 20–30 cm soil depth, respectively, for rubber-*A. villosum* intercropping system; CK1, CK2, CK3 = soil sampled at 0–10, 10–20, 20–30 cm soil depth, respectively, for monoculture rubber.

#### 3.1.3 Stoichiometry of soil C, N, and P

**Figure 1:**
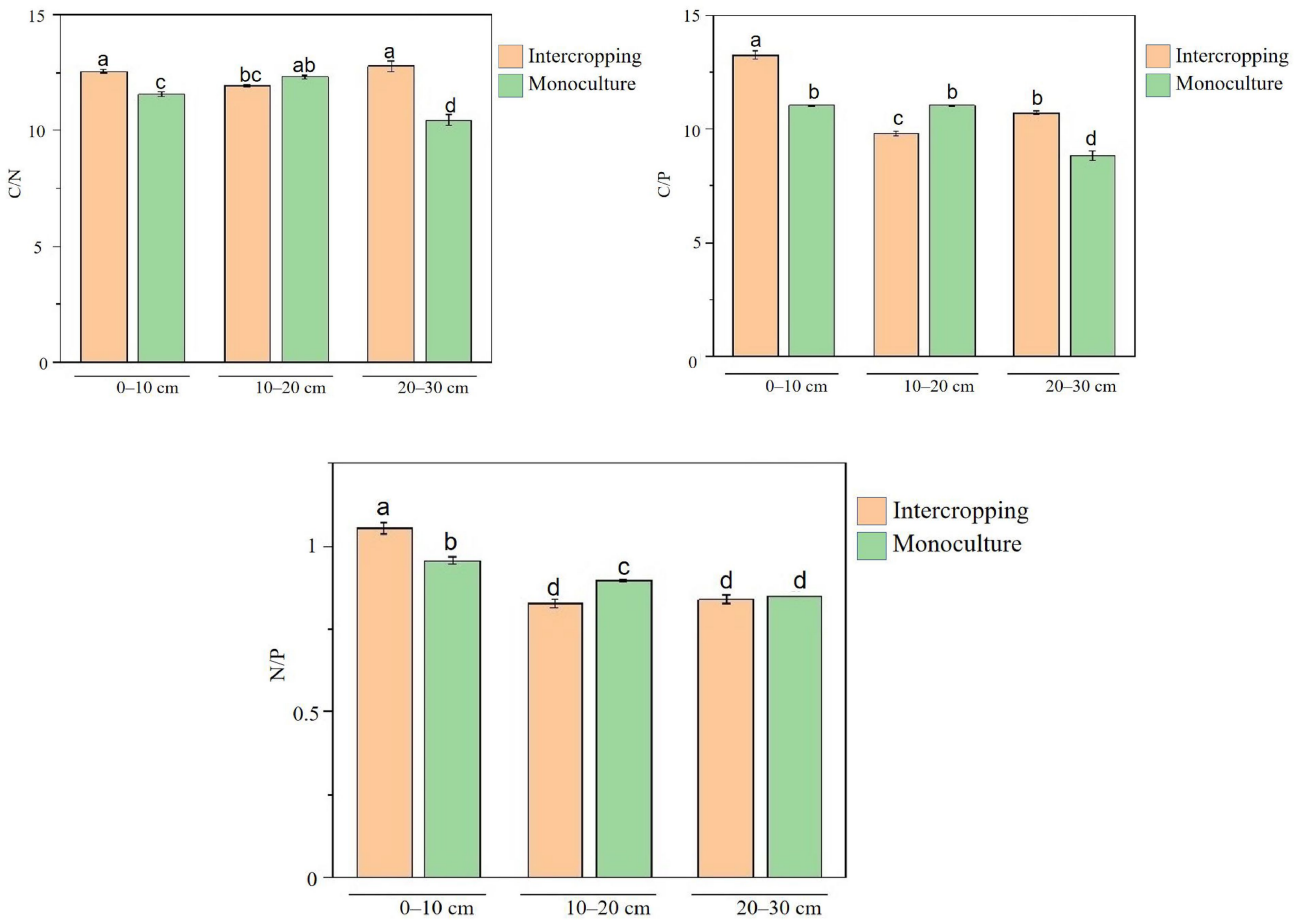
Stoichiometry of soil C, N, and P across depths under intercropped rubber-*Amomum villosum* and rubber monoculture. Bars show C/N, C/P, and N/P at 0–10, 10–20, and 20–30 cm (mean ± SD, *n* = 3). Different lowercase letters indicate significant differences at the *p* < 0.05 level (Tukey’s HSD test).

Under intercropping, the C/N ratio was significantly higher than in monoculture at 0–10 cm and 20–30 cm depths, with no difference at 10–20 cm (Figure 1). The N/P ratio increased for 0–10 cm but decreased for 10–20 cm, with no difference observed at 20–30 cm. C/P ratio was significantly higher at 0–10 cm and 20–30 cm depths under intercropping, whereas it was lower at 10–20 cm than in monoculture. These patterns align with the observed increases in SOM and AN at the surface and with the depth-linked N cycling.

### 3.2 Effects of intercropping on soil microbial diversity and community structure

#### 3.2.1 Sequence depth and alpha diversity

**Figure 2:**
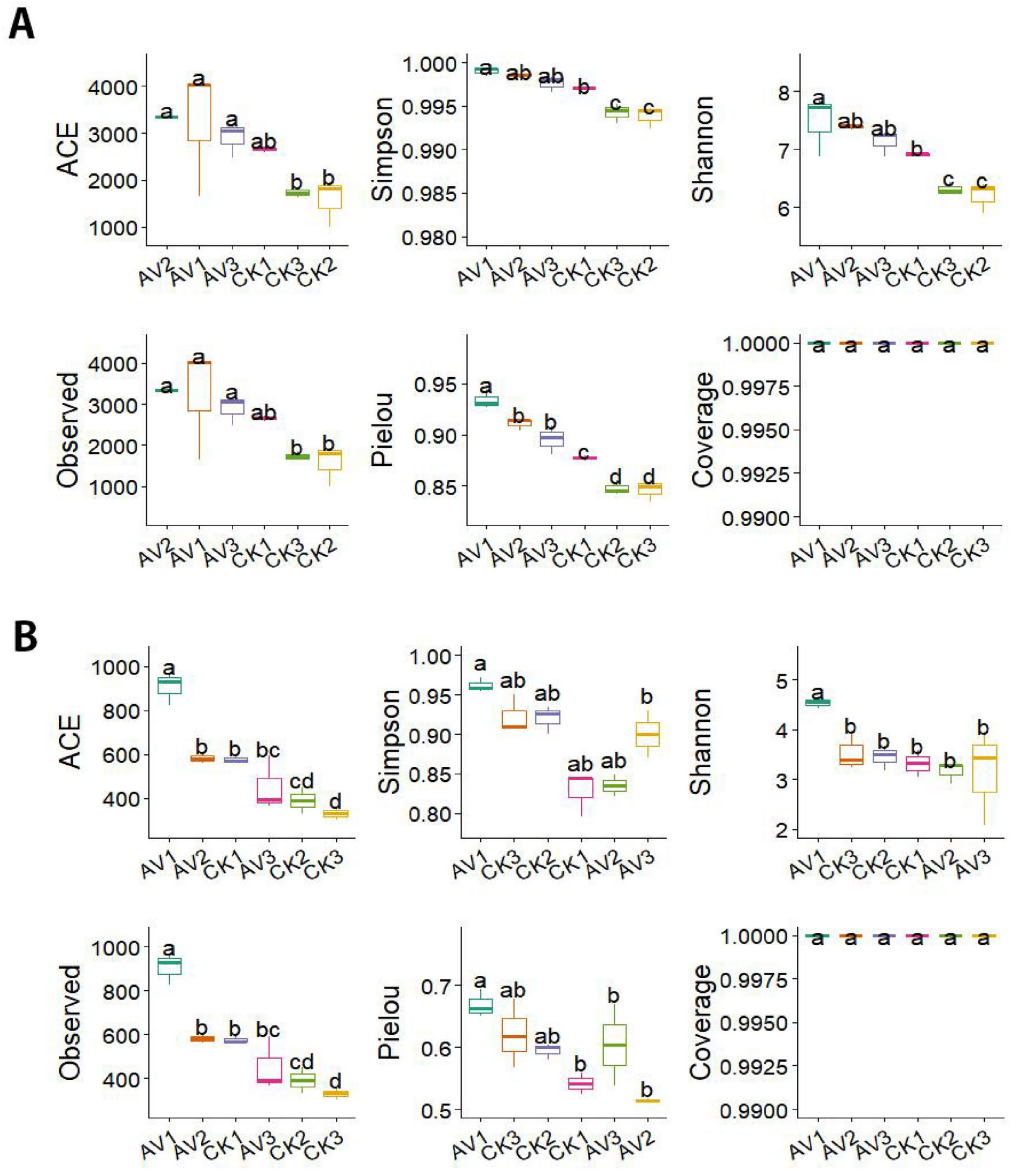
Alpha diversity of **(A)** bacterial and **(B)** fungal communities across soil depths under rubber-*Amomum villosum* intercropping and rubber monoculture. Panels showed the Observed, ACE, Shannon, Simpson, and Pielou indices (mean ± SD, *n* = 3). Different lowercase letters indicate significant differences at the *p* < 0.05 level (Tukey’s HSD after ANOVA). Sample codes: AV1–AV3 = intercropping at 0–10, 10–20, 20–30 cm; CK1–CK3 = monoculture at the same depths.

Sequencing depth was sufficient across all samples, with Good’s coverage approaching 1.0 (Supplementary Figure S2). Intercropping yielded consistently higher bacterial and fungal ASV richness than monoculture across depths. Alpha-diversity indices (Observed, ACE, Shannon, Simpson, Pielou) differed by both cropping system and soil depth (Figure 2). For bacteria, evenness (Pielou) was higher under intercropping at all depths. For fungi, diversity and evenness were significantly higher at 0–10 cm under intercropping, with no clear treatment differences at deeper layers. Overall, surface soils (0–10 cm) exhibited the strongest diversity responses, consistent with the larger edaphic changes observed in this layer.

#### 3.2.2 Beta diversity and community separation

Unweighted-UniFrac PCoA revealed clear separation of microbial communities by cropping system and by soil depth (Supplementary Figure S3). For bacteria, PCoA1 and PCoA2 explained 30.17 and 8.66% of variation, respectively, while fungi showed similar patterns (PCoA1 = 28.22%, PCoA2 = 11.02%). Intercropped and monoculture samples formed distinct clusters, and 0–10 cm communities were well separated from those at 10–20 and 20–30 cm, indicating strong depth-resolved structuring.

#### 3.2.3 Bacterial and fungal community composition

**Figure 3:**
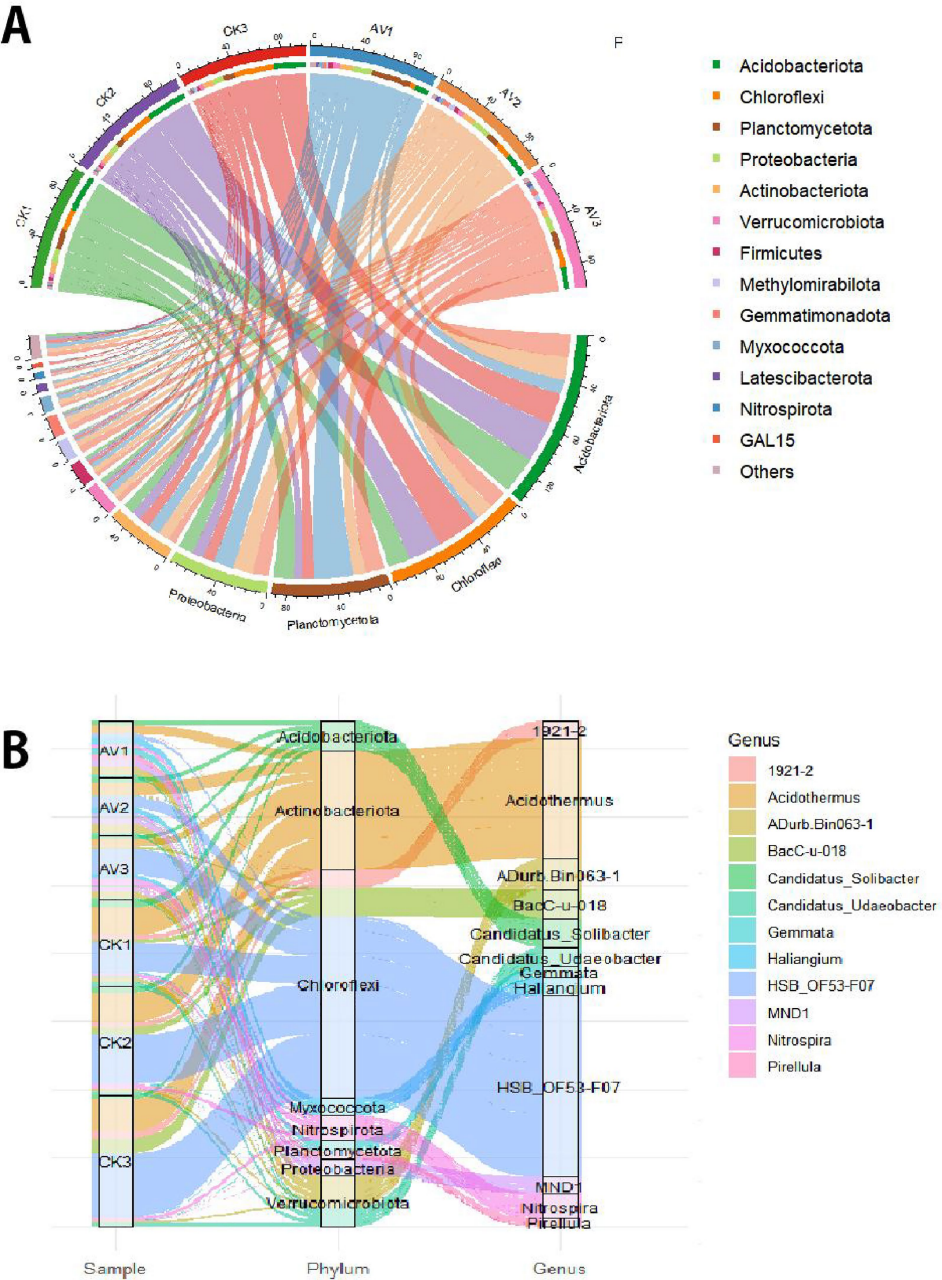
Bacterial community composition across depths under rubber-*Amomum villosum* intercropping and rubber monoculture. **(A)** Dominant bacterial phyla visualized using a chord diagram. **(B)** Key bacterial genera visualized with a Sankey diagram. Relative abundances are mean ± SD (*n* = 3). Sample codes: AV1–AV3 = intercropping at 0–10, 10–20, 20–30 cm; CK1–CK3 = monoculture at the same depths.

Intercropping maintained broader bacterial and fungal taxonomic breadth than monoculture (Supplementary Tables S1–S6). Dominant bacterial phyla included Acidobacteriota (11.8–30.8%), Chloroflexi (4.8–33.2%), Planctomycetota (7–32%), Proteobacteria (9.7–16.6%), and Actinobacteriota (6.2–10.2%) (Figure 3A). Intercropping increased the relative abundance of Proteobacteria, Planctomycetota, and Gemmatimonadota, while Acidobacteriota and Chloroflexi declined, particularly in surface soils. At the genus level, several groups linked to nutrient cycling or structured soils, such as *Haliangium*, *Nitrospira*, Candidatus_Solibacter, *Gemmata*, and *Pirellula,* were more abundant under intercropping (Figure 3B and Supplementary Tables S2, S3).

**Figure 4:**
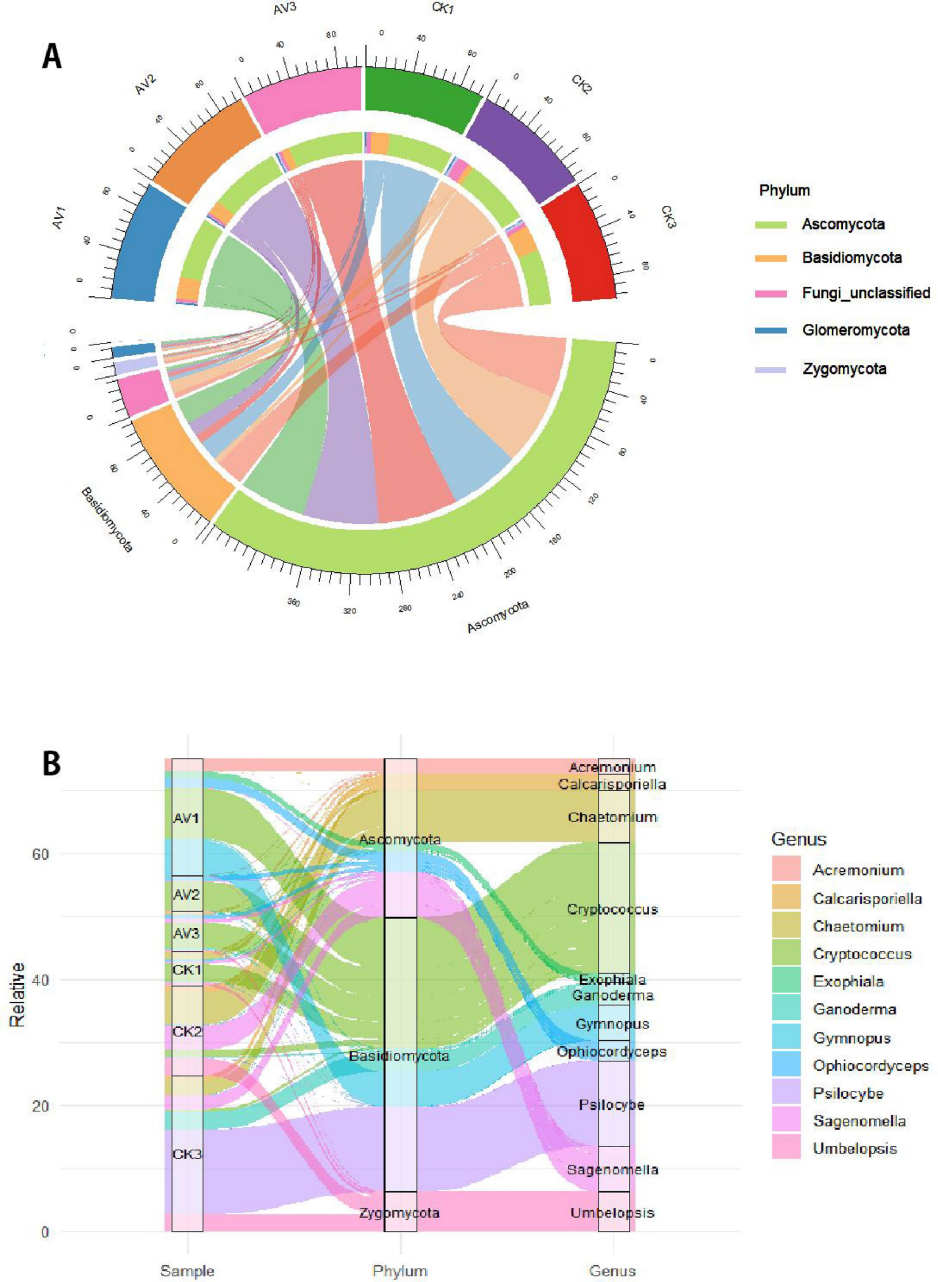
Fungal community composition across depths under rubber-*Amomum villosum* intercropping and rubber monoculture. **(A)** Dominant fungal phyla. **(B)** Dominant fungal genera. Relative abundances are mean ± SD (*n* = 3). Sample codes: AV1–AV3 = intercropping at 0–10, 10–20, 20–30 cm; CK1–CK3 = monoculture at the same depths. See Supplementary Tables S3, S4 for detailed values.

Fungal communities were dominated by Ascomycota and Basidiomycota, with Glomeromycota contributing modestly but increasing under intercropping (Figure 4A and Supplementary Table S4). Genera such as Ophiocordyceps, Exophiala, and Cryptococcus were enriched under intercropping, especially at 0–10 cm (Figure 4B and Supplementary Table S6). Depth-wise patterns indicated that symbiotrophic groups were more prominent in subsurface layers under intercropping.

#### 3.2.4 Depth and treatment-responsive taxa (LEfSe and ANOVA)

**Figure 5:**
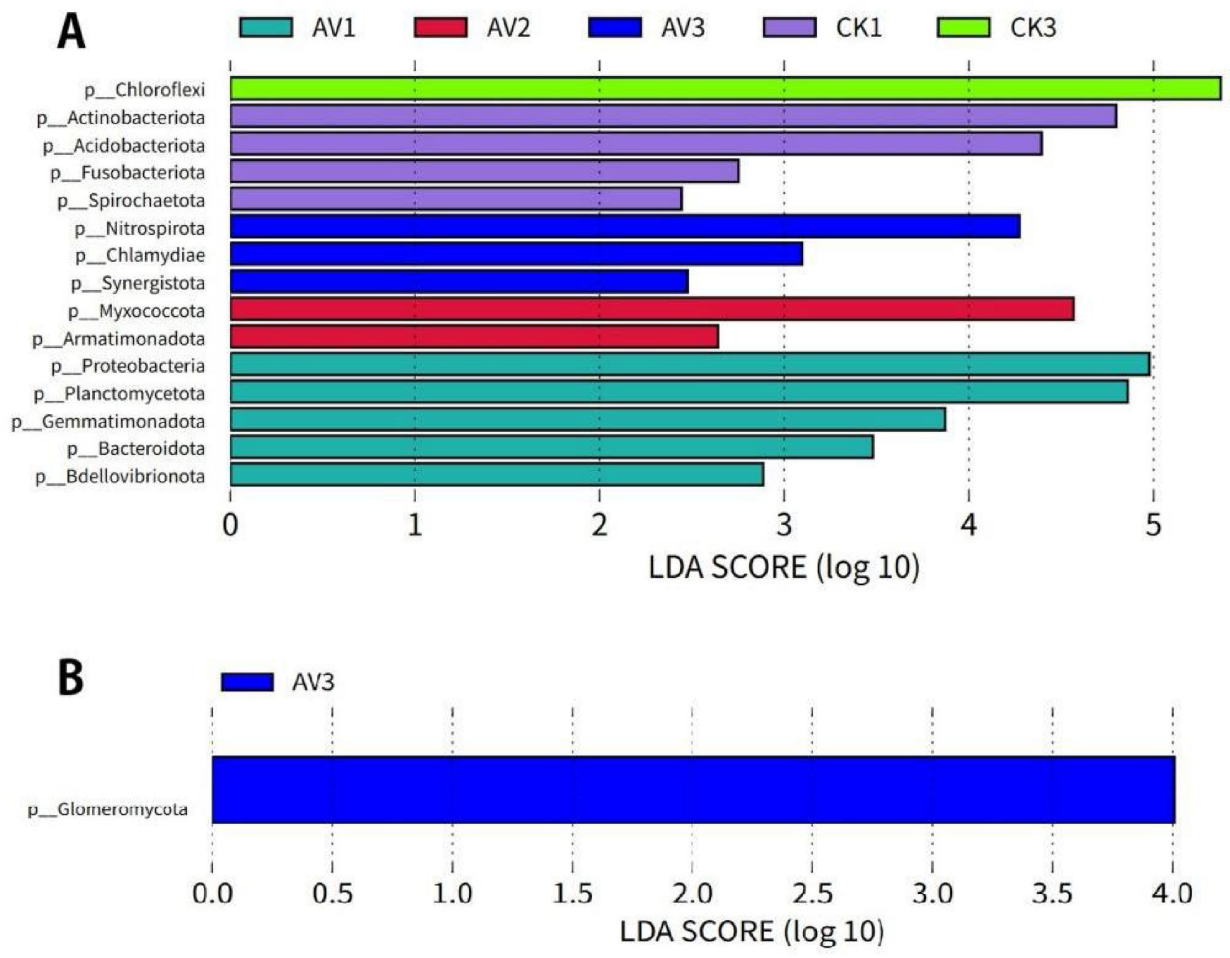
Phylum-level biomarkers identified by LEfSe (LDA >2). **(A)** Bacteria; **(B)** Fungi. Bars show LDA score for taxa with significant differential abundance between intercropping and monoculture across depths. LEfSe applies Kruskal-Wallis and Wilcoxon tests (*α* = 0.05) followed by LDA to estimate effect size. *n* = 3 per depth × treatment. Sample codes: AV1–AV3 = intercropping at 0–10, 10–20, 20–30 cm; CK1–CK3 = monoculture at the same depths.

**Figure 6:**
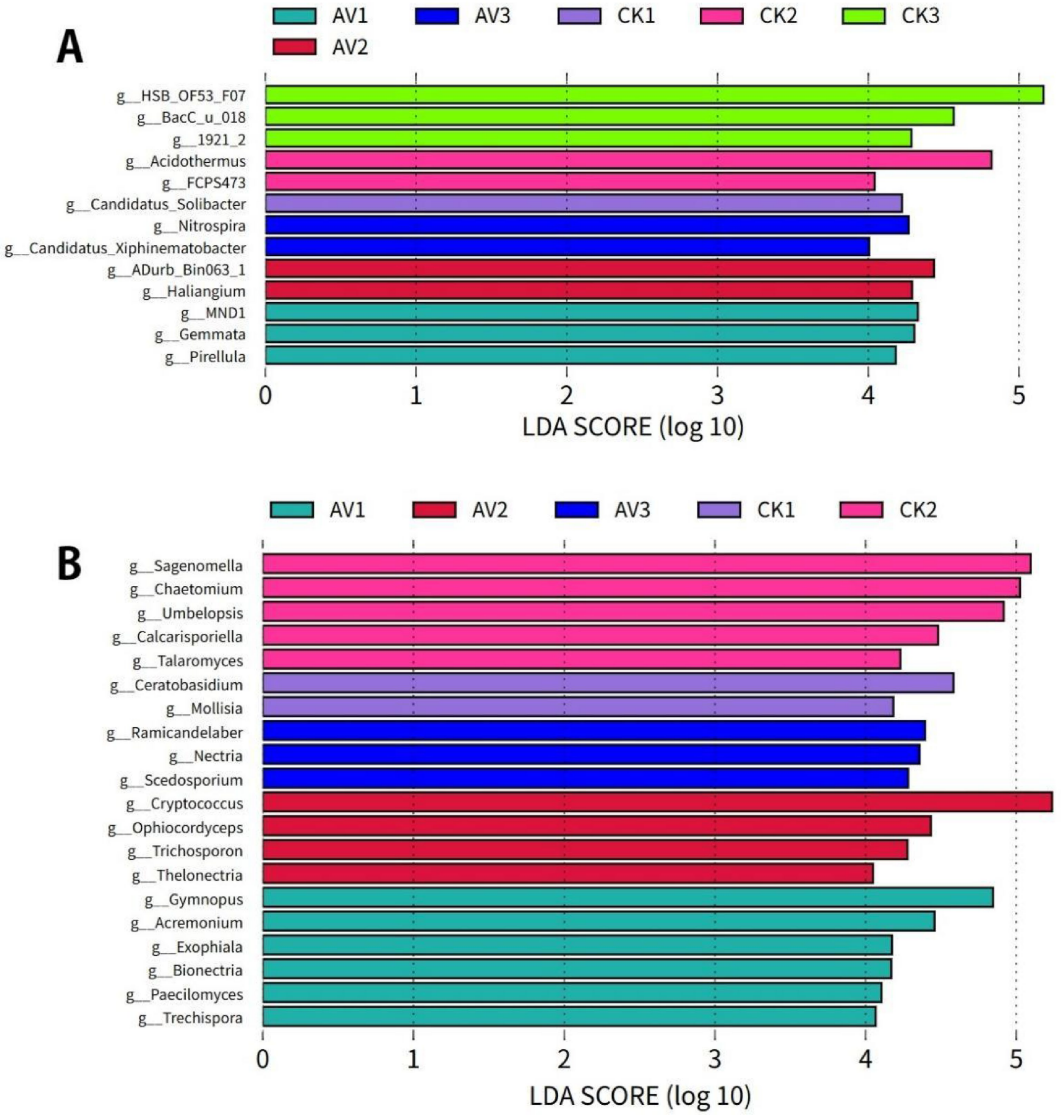
Genus-level biomarkers identified by LEfSe (LDA > 4). **(A)** Bacteria; **(B)** Fungi. Bars show LDA scores for genera with significant differential abundance between intercropping and monoculture across depths. LEfSe used Kruskal–Wallis and Wilcoxon tests (*α* = 0.05), followed by LDA to estimate effect size. *n* = 3 per depth × system. Sample codes: AV1–AV3 = intercropping at 0–10, 10–20, 20–30 cm; CK1–CK3 = monoculture at the same depths.

LEfSe identified distinct depth-specific bacterial and fungal biomarkers for each cropping system (Figures 5, 6). Under intercropping, Proteobacteria, Planctomycetota, and Gemmatimonadota were enriched at 0–10 cm, Myxococcota at 10–20 cm, and Nitrospirota at 20–30 cm. In contrast, monoculture soils were characterized by Acidobacteriota and Actinobacteriota at 0–10 cm and Chloroflexi at depth. Genus-level biomarkers under intercropping included MND1, Gemmata, Pirellula, Haliangium, and Nitrospira, while monoculture enriched groups such as Candidatus Solibacter, Acidothermus, and HSB_OF53-F07 (Figures 6A,B).

Two-way ANOVA supported these patterns: cropping system primarily influenced Proteobacteria and Glomeromycota, depth structured Nitrospirota and Glomeromycota, and interactions affected Acidobacteriota, Gemmatimonadota, and Myxococcota (Supplementary Tables S7–S9). These results indicate that community shifts reflect both intercropping and soil-profile stratification.

#### 3.2.5 Relationships between microbial groups and soil properties

**Figure 7:**
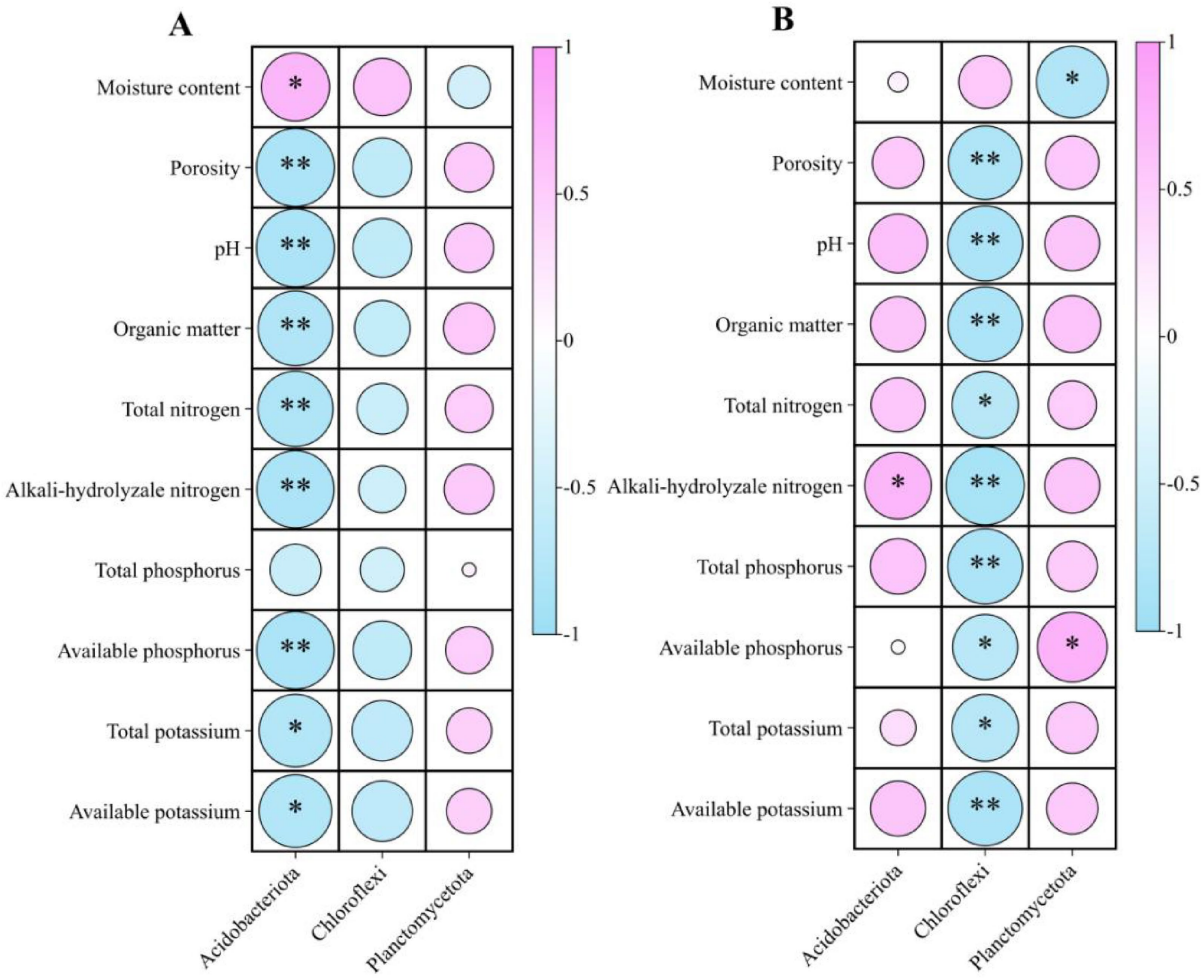
Spearman correlations (*ρ*) between bacterial phyla and soil properties. **(A)** rubber–*Amomum villosum* intercropping; **(B)** rubber monoculture. Circle color indicates the direction and magnitude of ρ (pink = positive, blue = negative; scale −1 to 1), and circle size is proportional to |ρ|. Asterisks indicate significance after multiple-test correction (Benjamini–Hochberg FDR): *q < 0.05; **q < 0.01; ns = not significant.

**Figure 8:**
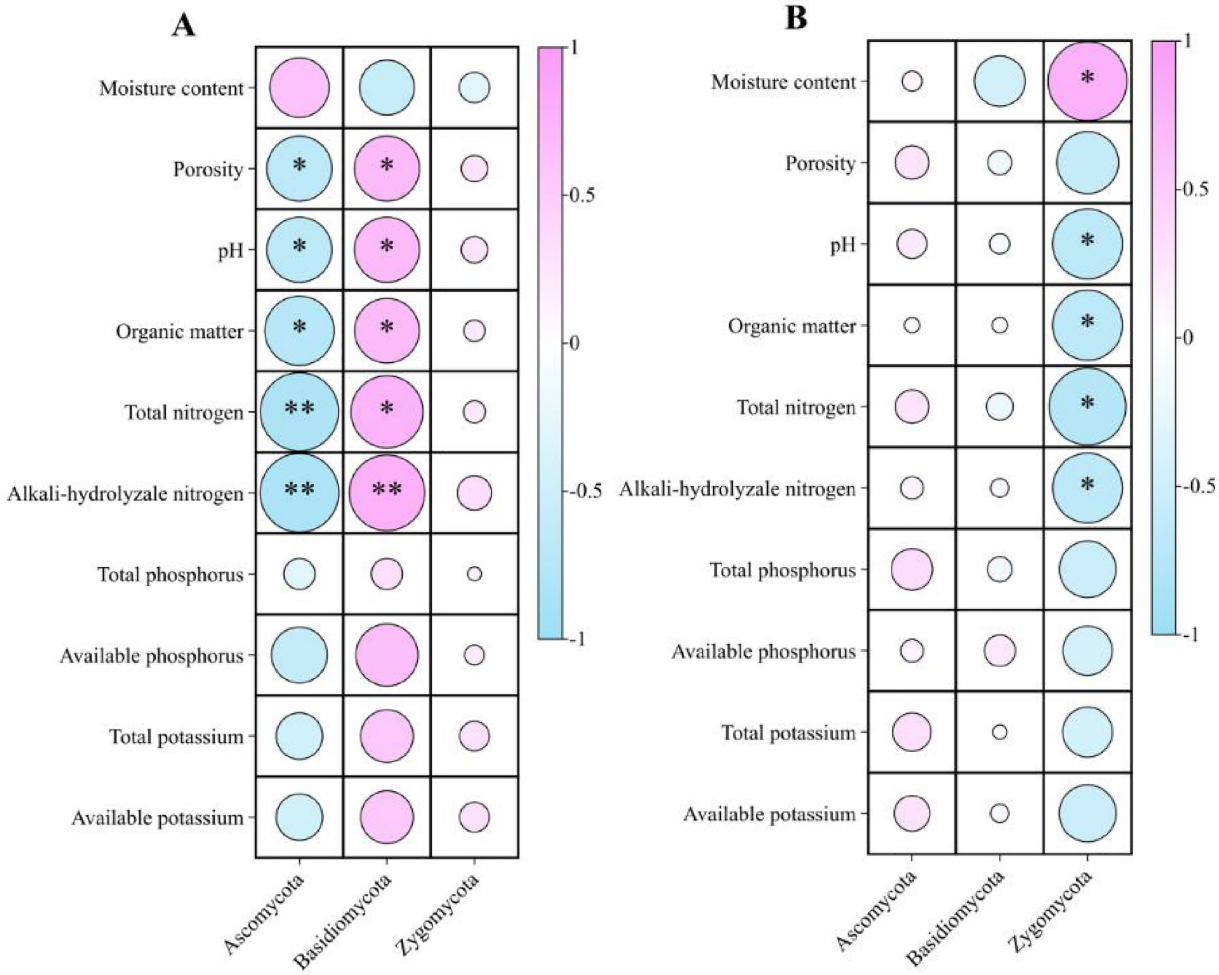
Spearman correlations between fungal phyla and soil properties. **(A)** rubber-*Amomum villosum* intercropping; **(B)** rubber monoculture. Circle color indicates the direction and magnitude of ρ (pink = positive, blue = negative; scale −1 to 1), and circle size is proportional to |ρ|. Asterisks indicate significance after multiple-test correction (Benjamini–Hochberg FDR): * *q* < 0.05; ** *q* < 0.01; ns = not significant.

Microbial–environment correlations revealed distinct phylum-level associations (Figures 7, 8). Under intercropping, Acidobacteriota correlated negatively with pH, SOM, TN, AN, and AP, whereas Basidiomycota correlated positively with these properties. Chloroflexi showed negative associations with most soil chemical variables. Planctomycetota was positively correlated with available P. In monoculture, Zygomycota correlated positively with moisture but negatively with SOM, TN, and AN. These patterns indicate that improvements in nutrient availability, pH, and soil organic matter under intercropping coincided with the enrichment of copiotrophic and symbiotrophic groups.

### 3.3 Effects of intercropping on predicted microbial functions

#### 3.3.1 Bacterial functional potentials (FAPROTAX)

**Figure 9:**
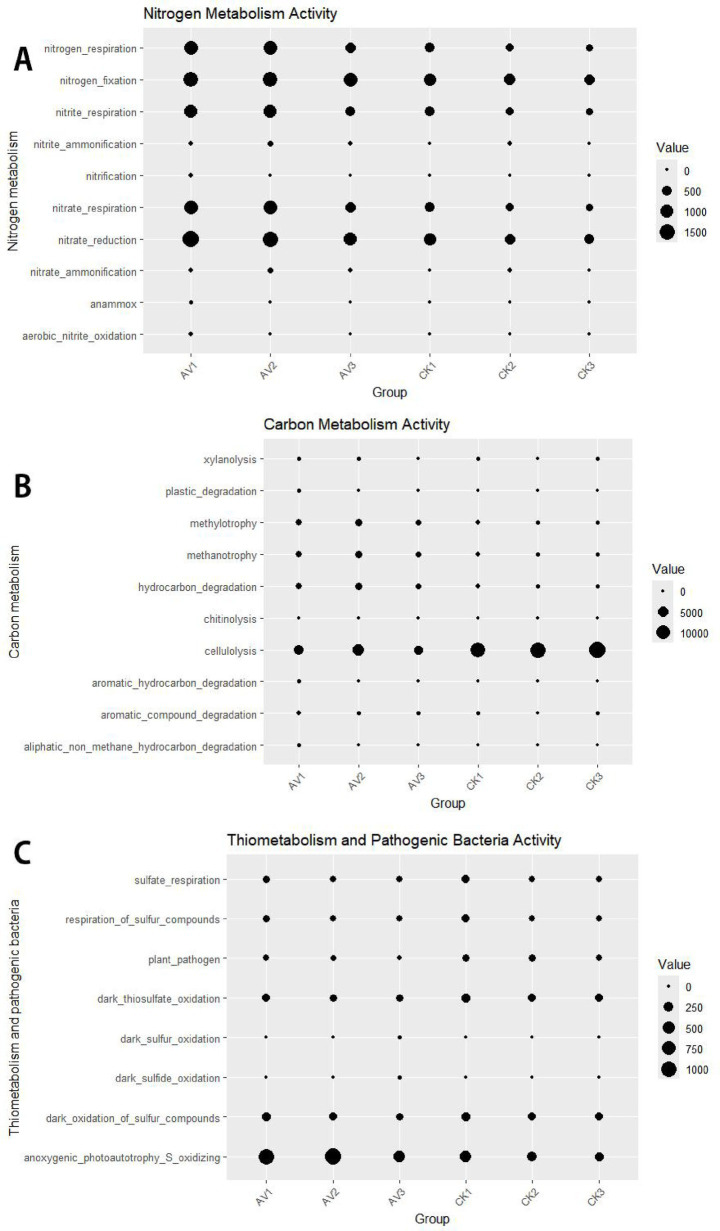
Predicted bacterial functions (FAPROTAX) across depths under rubber-*Amomum villosum* intercropping and rubber monoculture. **(A)** Nitrogen metabolism, **(B)** carbon metabolism, **(C)** sulfur metabolism. Bubble area is proportional to the cumulative relative abundance of ASVs annotated to each function; samples are grouped by depth and treatment (AV1–AV3 = intercropping at 0–10, 10–20, 20–30 cm; CK1–CK3 = monoculture at the same depths). Values are means across biological replicates (*n* = 3). Functions are predictions based on taxonomic assignment (FAPROTAX).

Functional predictions revealed consistent shifts in bacterial metabolic capacities under intercropping (Figure 9). Pathways associated with nitrogen fixation, nitrification, and nitrate/nitrite reduction increased across all depths, aligning with the enrichment of genera such as Bradyrhizobium, Azovibrio, and Nitrospira (Supplementary Figure S6). Intercropping also enhanced predicted methylotrophy, methanotrophy, hydrocarbon degradation, and xylanolysis, while reducing predicted cellulolysis and bacterial “plant_pathogen” categories. These changes reflect improved organic substrate availability and reduced pathogen pressure in intercropped soils.

#### 3.3.2 Fungal trophic modes and guilds (FUNGuild)

**Figure 10:**
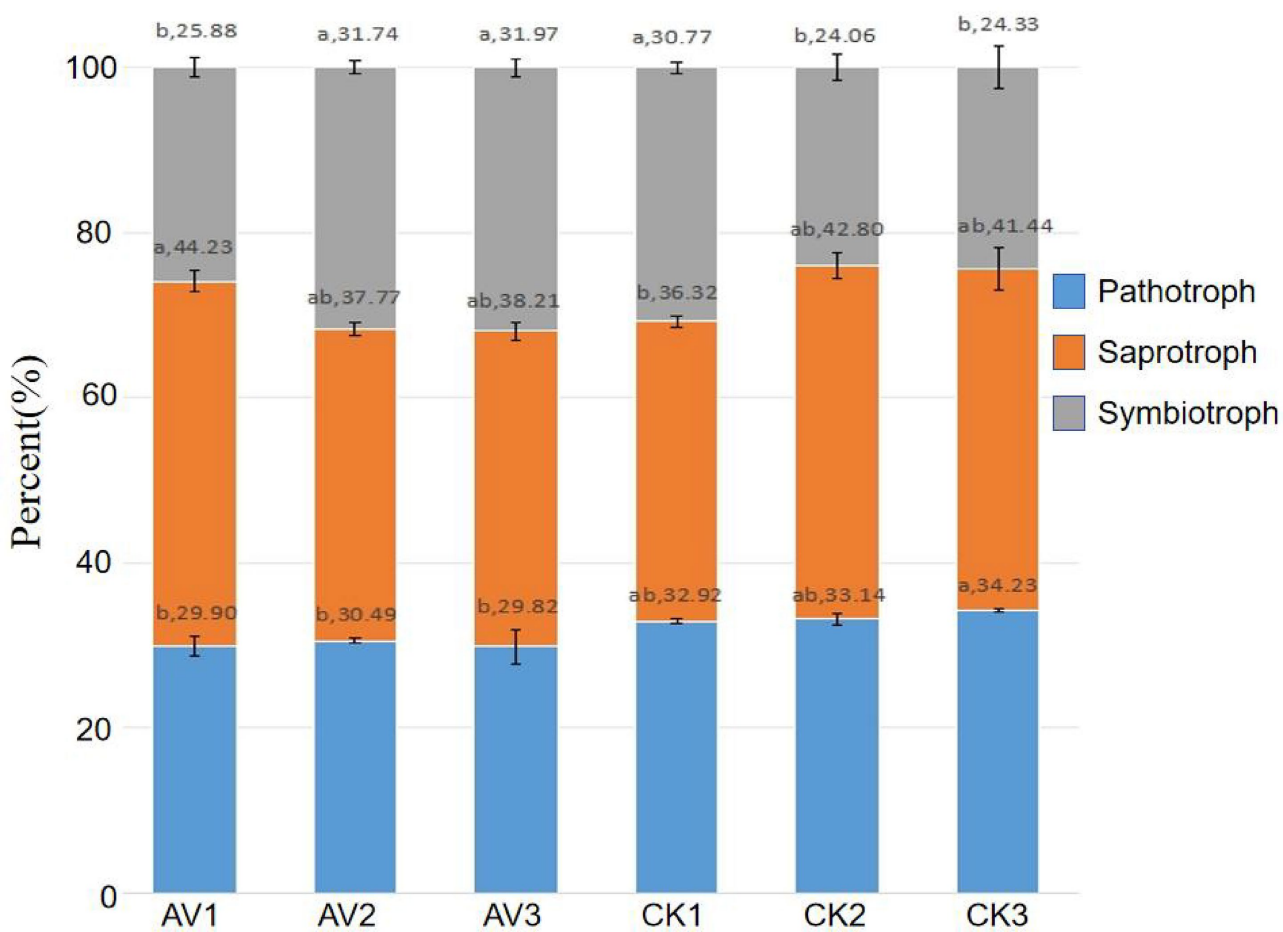
Predicted fungal trophic modes (FUNGuild) across treatments and depths. Proportional abundance of saprotroph, symbiotroph, and pathotroph categories for AV1–AV3 (intercropping at 0–10, 10–20, 20–30 cm) and CK1–CK3 (monoculture, same depths). Different lowercase letters within a depth indicate *p* < 0.05 (two-way ANOVA on arcsine-square-root–transformed proportions with Tukey’s HSD). Values are mean ± SD (*n* = 3).

Fungal guild predictions showed a shift from pathotroph-dominated communities in monoculture toward greater symbiotroph representation under intercropping (Figure 10). The 0–10 cm intercropped layer showed moderate pathotroph presence, but 10–20 cm and 20–30 cm layers were dominated by symbiotrophs, including arbuscular mycorrhizal fungi (Glomeromycota). Monoculture soils retained higher proportions of plant pathogens across depths. Supplementary analyses (Supplementary Figure S7 and Supplementary Table S10) confirmed the enrichment of mutualistic taxa, such as Glomeromycota and Ophiocordyceps, under intercropping. These patterns indicate that intercropping promotes fungal communities associated with nutrient acquisition and ecosystem functioning.

## 4 Discussion

### 4.1 Overview of soil depth-induced responses

Intercropping improved soil structure and nutrient status in ways consistent with earlier agroforestry research (Cuartero et al., 2022; Zhao et al., 2022; Gao and Xie, 2023; He et al., 2023; Tong et al., 2024). Across depths, soil under *Amomum villosum* intercropping system showed high porosity, greater moisture retention, and enhanced concentrations of soil organic matter (SOM), total N (TN), total P (TP), total K (TK), available P (AP), and alkali-hydrolysable N (AN), compared with monoculture. These effects were most pronounced in the 0–10 cm layer, but improvements extended into deeper layers.

Microbial communities responded in parallel: bacterial and fungal alpha diversity increased under intercropping, particularly at the soil surface (0–10 cm), and beta-diversity analyses showed clear separation of intercropped and monoculture communities across depths. Shifts in bacterial composition included increases in Proteobacteria, Planctomycetota, and Gemmatimonadota, with decreases in Acidobacteriota and several Chloroflexi. Depth-wise enrichment of Nitrospirota further aligned with nitrogen availability patterns. Fungal communities under intercropping showed higher representation of symbiotrophic groups at depth and reduced pathotroph signals in the deepest layer. Functional predictions indicated increased potential for nitrogen transformations, methylotrophy and methanotrophy, xylanolysis, and sulfur cycling, alongside reduced signals for cellulolysis and bacterial “plant_pathogen” categories. These depth-resolved responses are consistent with prior intercropping findings in both general agricultural settings and rubber-based systems (Luo et al., 2016; Li et al., 2018; Tang et al., 2022; Zhang et al., 2023; Qi et al., 2024).

### 4.2 Soil structure and water retention

The soil physical properties showed improvement with intercropping. The most substantial physical improvements occurred in the surface layer, where fine roots and litter inputs of *A. villosum* are maximum. Increased porosity at 0–10 cm suggests enhanced aggregation and biopore formation, a process frequently reported in intercropping and agroforestry systems (Lu et al., 2023; Andriyana et al., 2020). Higher moisture at 20–30 cm in intercropped soils aligns with documented complementary water use patterns in rubber agroforestry systems, where intercrops use shallow moisture while rubber relies more on deeper reserves, reducing direct competition and improving soil-profile water capture (Wu et al., 2016, 2019; Andriyana et al., 2020). The simultaneous increase in porosity and maintenance of higher moisture at depth suggests improved pore connectivity and storage, conditions that favor stable microbial habitats.

### 4.3 Nutrient responses and stoichiometry patterns

Increases in SOM, AP, and AN under intercropping reflect improved organic inputs and nutrient turnover, consistent with previous intercropping studies (Luo et al., 2016; Li et al., 2018; Tang et al., 2022). In rubber systems specifically, similar improvements in carbon and nutrient stocks have been reported under interplanted *Acacia chinensis* and *Pandanus amaryllifolius* (Qi et al., 2024; Zhang et al., 2023). Our observation that AN increased more than TN across depths suggests enhanced short-term nitrogen availability rather than simple accumulation of total N. Supporting mechanisms include higher SOM inputs, better physical protection of organic N in aggregated soils (Peng et al., 2023), and the increased presence of microbial groups associated with nitrogen cycling. Slight increases in soil pH—while remaining within an acidic range—also support more active decomposition and mineralization processes (Luo et al., 2016; Li et al., 2018; Tang et al., 2022). Increased AP likely reflects several processes acting together—greater organic inputs, phosphatase activity associated with mycorrhizal fungi, and enhanced sorption–desorption buffering as aggregates develop (Qu et al., 2024; Zhou et al., 2025).

### 4.4 Microbial diversity and composition in context

Higher microbial diversity and evenness under intercropping were strongest at 0–10 cm, mirroring the depth profile of soil improvements. Community shifts were consistent with resource conditions: copiotrophic Proteobacteria increased with higher SOM and porosity (Zheng et al., 2022; Song et al., 2024). In contrast, Acidobacteriota and parts of Chloroflexi, often associated with acidic or nutrient-poor soils, declined as resource availability increased (Lu et al., 2023; Peng et al., 2023; Tang et al., 2024). Our correlation analyses further support these responses, showing negative associations between Acidobacteriota and key chemical properties under intercropping, whereas Basidiomycota showed positive associations with SOM and N (see also Ye et al., 2019; Tang et al., 2024). The depth-wise rise of Nitrospirota, including nitrifying *Nitrospira*, aligns with higher AN and more stable, oxic conditions at depth, where nitrification typically proceeds (Noll et al., 2005; Yu et al., 2023). LEfSe biomarkers reinforced these patterns, indicating depth-specific enrichment of *Nitrospira* (N cycling) and Planctomycetota (e.g., *Gemmata*, *Pirellula*) that often associate with particle-attached lifestyles, as well as *Haliangium* (Myxococcota), a predatory lineage frequently reported in structured, organic-rich soils (Peng et al., 2023).

### 4.5 Functional potentials from FAPROTAX and FUNGuild

Functional predictions were consistent with observed taxonomic changes and soil properties. Increased potentials for nitrogen fixation, nitrification, and nitrate/nitrite transformations matched both the rise in AN and the enrichment of relevant bacterial lineages such as *Bradyrhizobium*, *Azovibrio*, and *Nitrospira* (Lian et al., 2019; Shu et al., 2024; Lu et al., 2025). Enhanced methylotrophy, methanotrophy, hydrocarbon degradation, and xylanolysis reflect the improved aeration and chemical diversity of intercropped soils. Declines in cellulolysis paralleled the reduced abundance of Acidothermus, a cellulolytic genus (Wang et al., 2024; Tang et al., 2024). For fungi, FUNGuild indicated a surface-to-subsurface rebalancing of trophic modes: fewer pathotrophs at 20–30 cm and more symbiotrophs (including arbuscular-mycorrhizal Glomeromycota) at 10–30 cm in intercropped soils. These patterns, together with biomarker enrichment of endophytic or insect-associated *Ophiocordyceps* (Sikorski et al., 2022), align with a belowground community structure that is more compatible with nutrient acquisition and potentially lower disease pressure.

### 4.6 Rhizosphere inputs and near-surface diversity

Although root exudates were not directly measured, the strong surface-layer responses observed here align with the role of rhizosphere carbon inputs reported for *A. villosum* and other intercrops. Existing studies show that *A. villosum* roots release organic acids, sugars, and amino acids that selectively enrich copiotrophic bacteria and certain fungi (Hawes et al., 2002; Ye et al., 2019; Li et al., 2022). Prior work on *A. villosum* also reports relative enrichment of Proteobacteria and Actinobacteria with suppression of Acidobacteriota in its rhizosphere (Du et al., 2025), a pattern consistent with the present community shifts. These considerations help explain why alpha-diversity responses were most significant at 0–10 cm, where fine roots and litter inputs are most concentrated.

Taken together, these depth-resolved soil and microbiome responses address the hypotheses stated in the Introduction. The improvements in porosity, moisture, SOM, and nutrient availability under intercropping support our expectation that soil structure and nutrient status would be enhanced. The increases in bacterial and fungal alpha diversity, along with depth-dependent shifts in community composition, are consistent with the predicted changes in microbial richness and stratification. Likewise, the enrichment of nitrogen-cycling lineages, declines in cellulolytic and pathogenic groups, and increases in symbiotrophic fungi align with the anticipated enhancement of functional potentials for nutrient cycling. Finally, the clear depth × treatment patterns observed across physical, chemical, and biological indicators confirm that intercropping effects vary with soil depth in ways that reflect complementary resource use by rubber and *A. villosum*.

### 4.7 Ecological implications and practical considerations

Intercropping improved physical structure, moisture dynamics, and nutrient availability in ways that enhance rooting conditions for rubber. These benefits extended through the soil profile, with the strongest gains in porosity, SOM, AN, and AP at 0–10 cm and measurable improvements down to 20–30 cm. The accompanying shift in microbial communities—toward copiotrophic bacteria, symbiotrophic fungi, and predatory groups such as Haliangium, and away from fungal pathotrophs—reflects a belowground environment that is more supportive of nutrient acquisition and potentially lower disease pressure. These patterns align with previous reports of intercropping-mediated disease suppression and soil health improvements (Ye et al., 2019; Torres-Bedoya et al., 2025) and reinforce the agronomic appeal of *A. villosum* in rubber plantations (Cuartero et al., 2022; Zhao et al., 2022; Tong et al., 2024).

Together, these depth-resolved changes suggest practical management strategies that can be readily adopted. Maintaining surface residues helps preserve the structural and nutrient benefits concentrated in the topsoil. Adjusting nitrogen application to account for elevated AN under intercropping can prevent unnecessary inputs, while minimizing soil disturbance supports the continuity of arbuscular mycorrhizal fungi in subsurface layers. Implementing these practices can enhance nutrient capture efficiency and promote more stable soil health, reinforcing the suitability of rubber–*A. villosum* intercropping as a sustainable management option in tropical perennial systems.

### 4.8 Limitations and outlook

Two limitations warrant emphasis. First, amplicon sequencing and function-prediction tools (FAPROTAX, FUNGuild) indicate potential functions rather than measured process rates. Second, our sampling represents a single season at one site. Future work should link community patterns to process measurements (net N mineralization and nitrification, phosphatase activity), quantify arbuscular-mycorrhizal colonization, and assess hydrolytic enzyme activities, while validating functional capacities with shotgun metagenomics or metatranscriptomics. Multi-year, multi-site trials are needed to evaluate durability across climates and management regimes, and to relate soil changes to rubber yield and latex quality directly. Finally, quantifying root traits (biomass profiles, rooting depth, exudation) and aggregate stability would further connect plant strategies, soil structure, and microbial composition.

## 5 Conclusion

Intercropping *A. villosum* with rubber has optimized the physical and chemical properties of the rubber forest soil as a whole by improving soil physical structure, regulating chemical properties, and enhancing nutrient availability. This improvement is most significant in the shallow soil layer (0–10 cm). Meanwhile, intercropping *A. villosum* significantly enhanced the diversity and richness of soil bacteria and fungi in rubber plantations, particularly in the shallow soil layers, where the effect was more pronounced. This planting model enhances the nutrient absorption capacity of rubber roots, forming a nutritionally complementary intercropping system suitable for promotion and planting under rubber forests.

## Data Availability

Raw sequencing data in FASTQ format were publicly available via the NCBI Sequence Read Archive (BioProject ID: PRJNA1372903).
