# Supplementary material for: Intercropping Amomum villosum enhances soil stratification, nutrient complementarity, and microbial communities in rubber plantations
Source: Front Microbiol. 2026 Jan 7;16:1720828. doi: 10.3389/fmicb.2025.1720828 (PMC12819771; doi:10.3389/fmicb.2025.1720828)
Supplement: Supplementary file 1 [file Data_Sheet_1.docx]

Supplementary Material


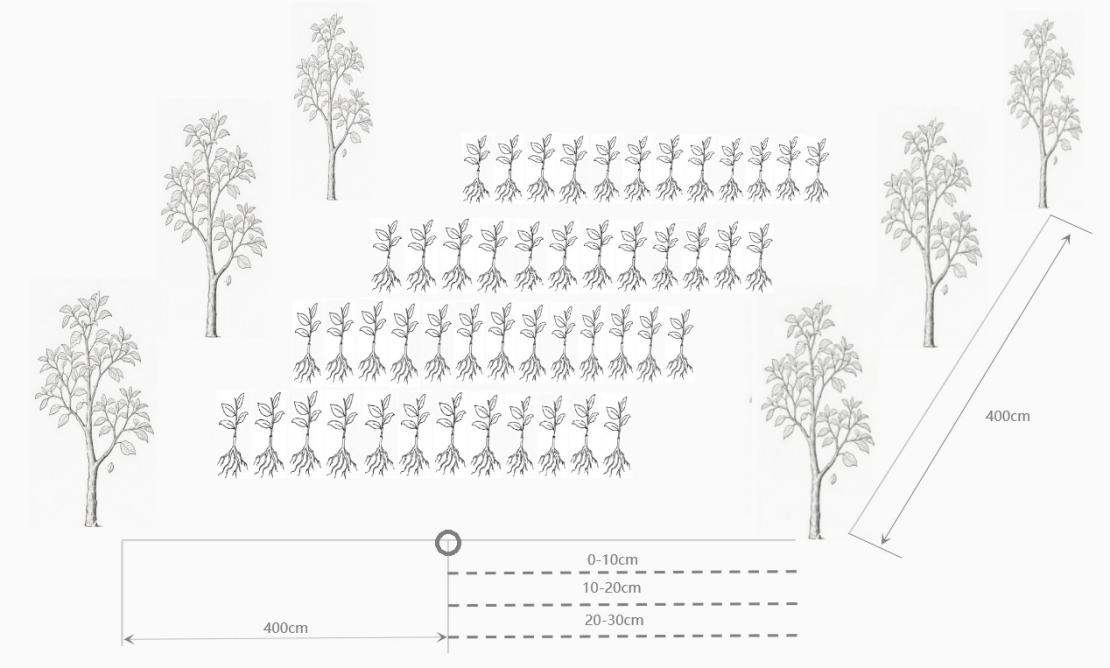


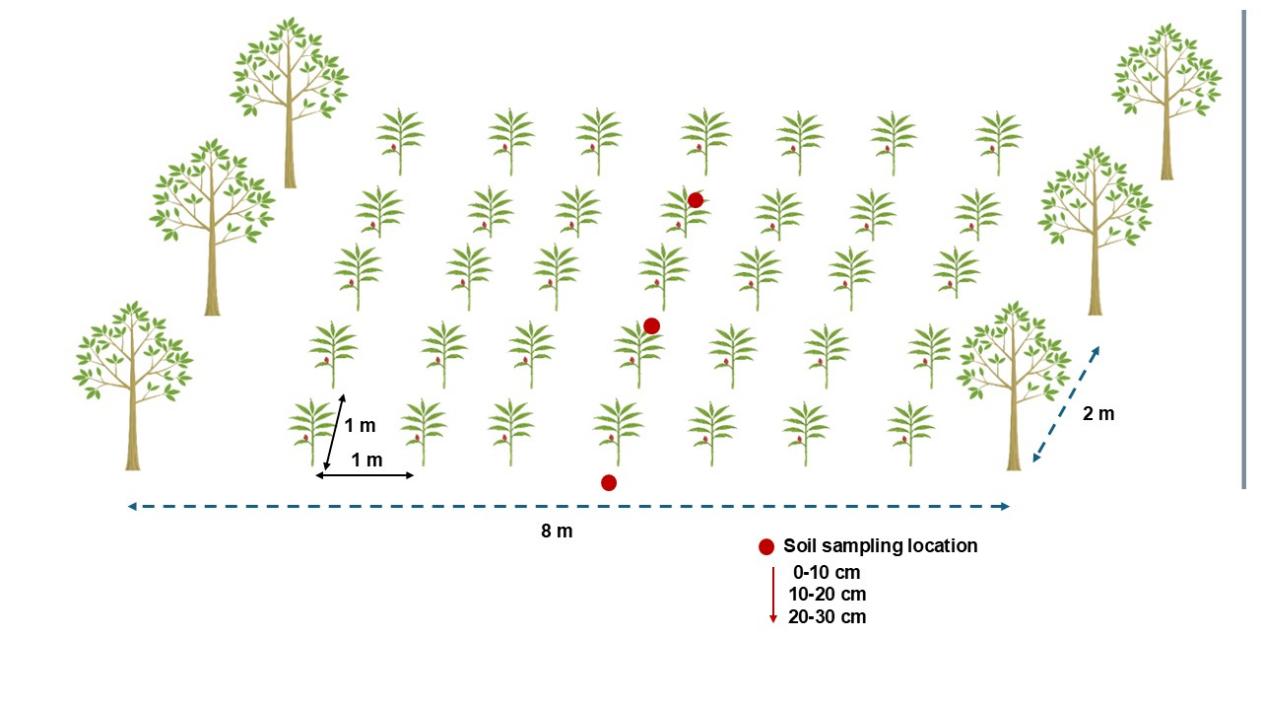


**Figure S1 Planting geometry and sampling layout for rubber–*Amomum villosum* intercropping.** Red dots indicate sampling point positioned mid row (4 m from rubber rows); stratified depths: 0–10, 10–20, 20–30 cm; n = 3 per depth x system.


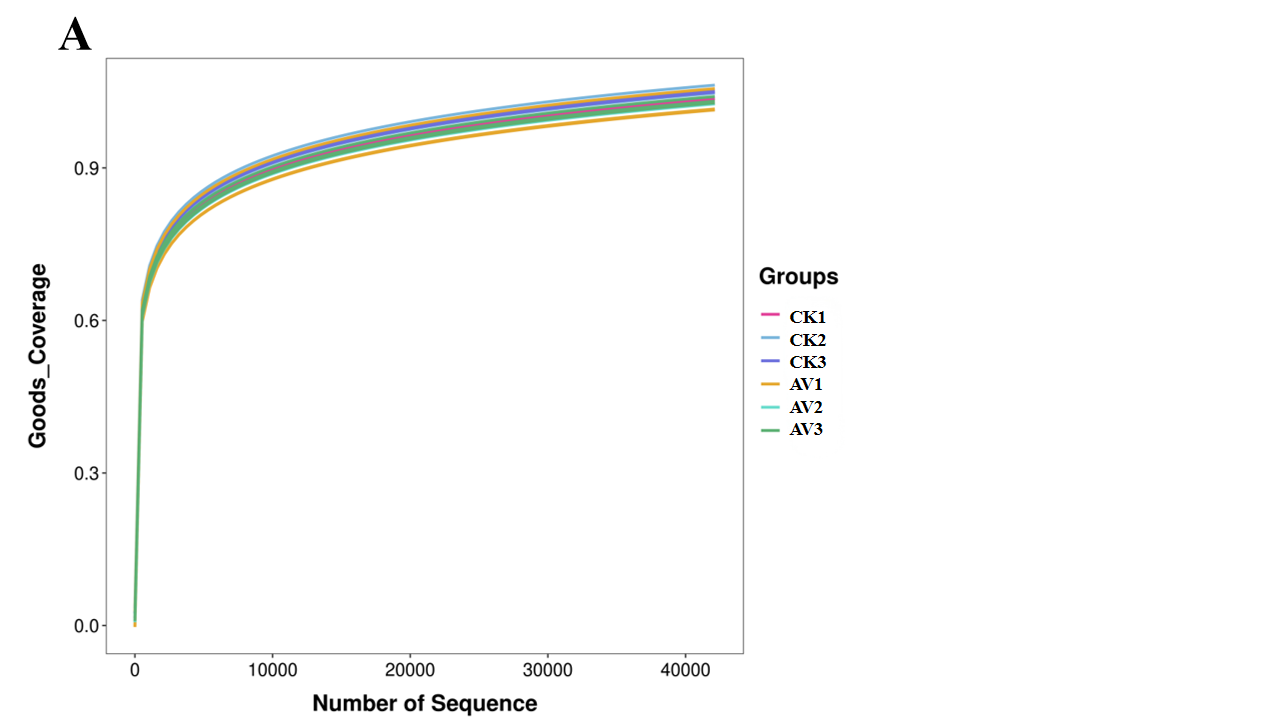

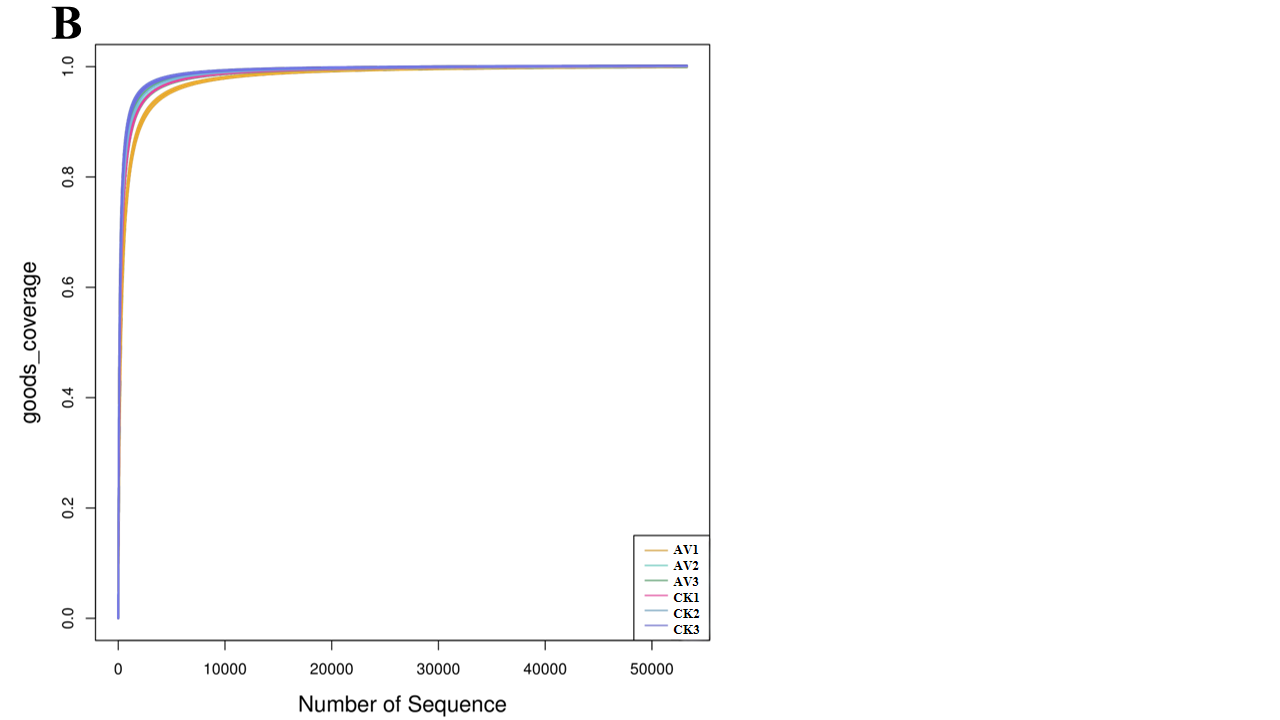


Figure S2 Rarefaction curves. (A) bacteria; (B) fungi. Sample codes: AV1–AV3 = intercropping at 0–10, 10–20, 20–30 cm; CK1–CK3 = monoculture at the same depths.


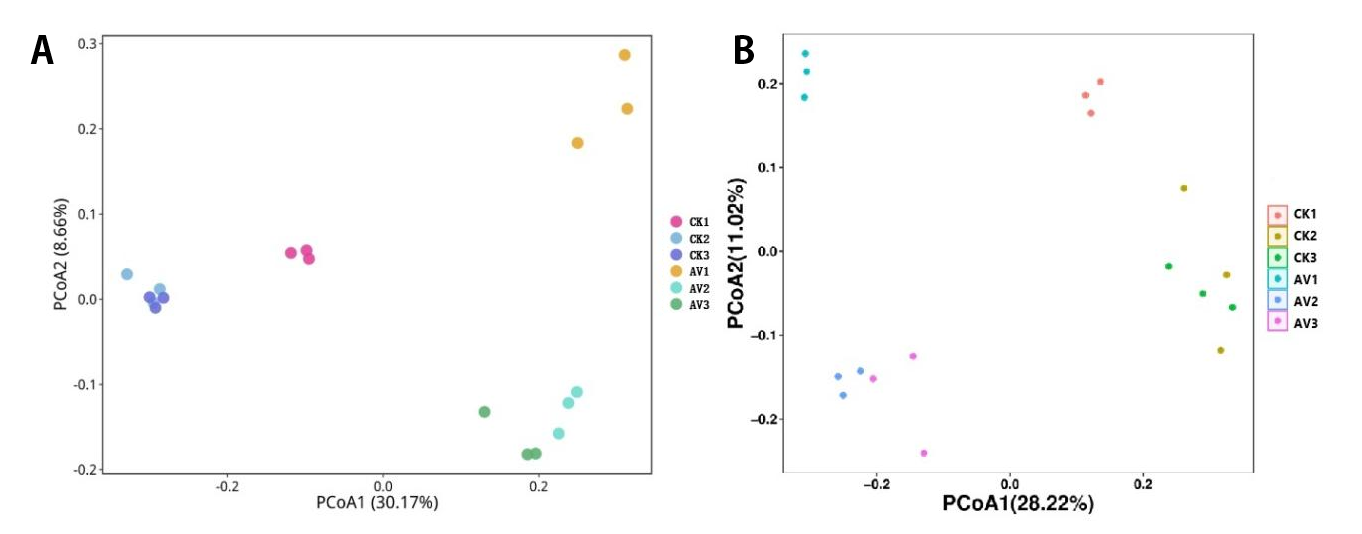


**Figure S3 Principal coordinate analysis (PCoA, unweighted-UniFrac) of (A) bacteria and (B) fungi.** Ellipses show 95% confidence regions by treatment, and axes show the variance explained.


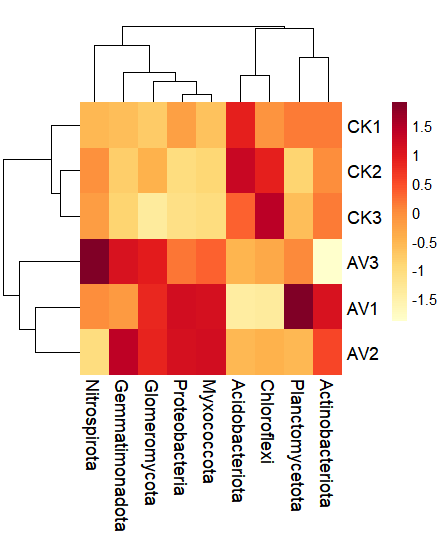


**Figure S4 Heat map of dominant bacterial phyla across treatments and depths.** Cells show row-scaled (z-score) log10 relative abundances; rows (phyla) and columns (samples) are hierarchically clustered (Euclidean distance, complete linkage). Sample codes: AV1–AV3 = intercropping at 0–10, 10–20, 20–30 cm; CK1–CK3 = monoculture at the same depths. Color scale −2 to +2 (lighter = lower, darker = higher). Values are means of biological replicates (n = 3).


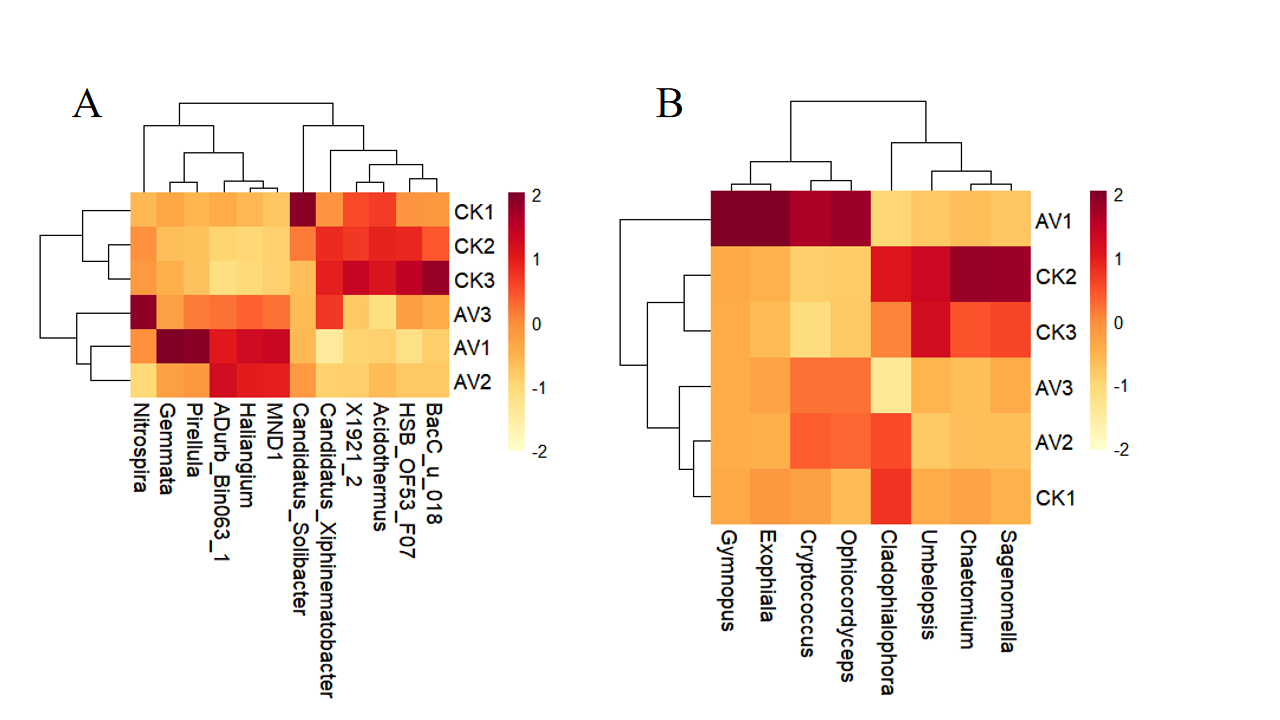


**Figure S5 Heat map of dominant genera across treatments and depths.** (A) Bacteria; (B) Fungi. Cells show row-scaled (z-score) log10 relative abundances; rows (genera) and columns (samples) are hierarchically clustered. Sample codes: AV1–AV3 = intercropping at 0–10, 10–20, 20–30 cm; CK1–CK3 = monoculture at the same depths.


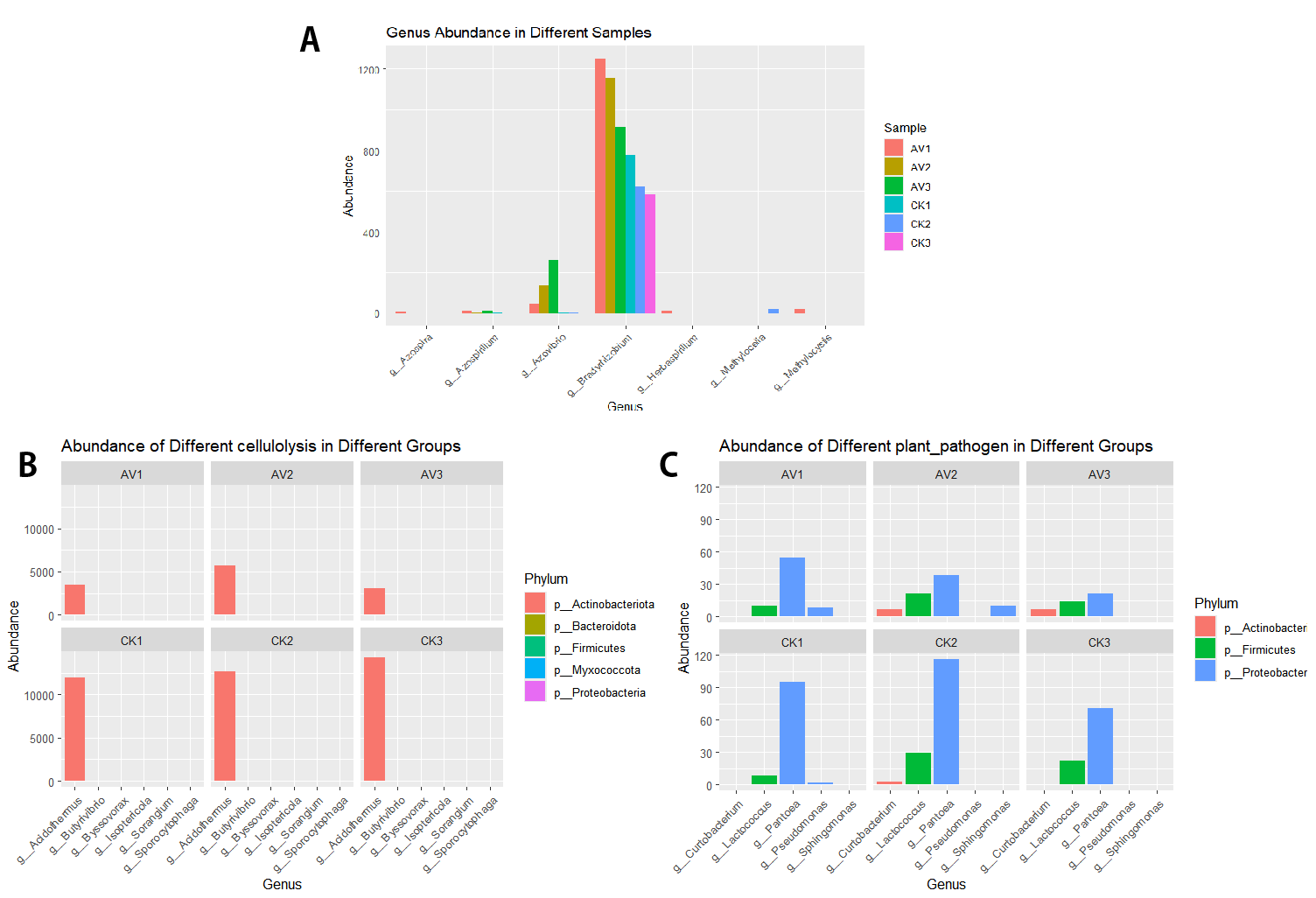


**Figure S6 Genera underpinning selected FAPROTAX categories.** (A) Putative nitrogen-fixing genera (e.g., *Bradyrhizobium*, *Azovibrio*). (B) Cellulose-degrading genera (e.g., *Acidothermus*). (C) Genera within the plant_pathogen category (e.g., *Pantoea*, *Lactococcus*). Bars show relative abundance by sample (AV1–AV3, CK1–CK3); facet headers indicate depth groups. Colors denote phylum. Values are means of n = 3 replicates per group.


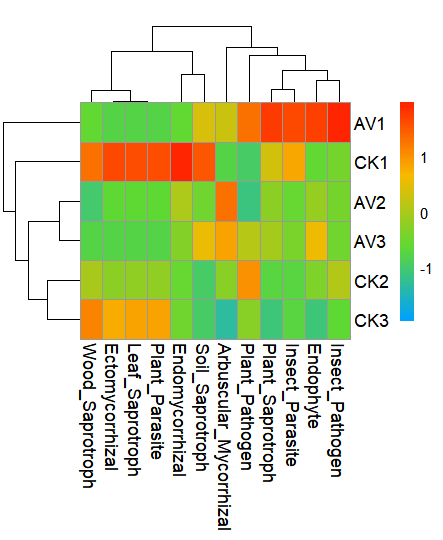


**Figure S7. Heat map of fungal ecological guilds.** Guilds (rows) and samples (columns) are hierarchically clustered (Euclidean distance, complete linkage) using row-scaled (z-score) log10 relative abundances. Intercropped AV1 is associated with higher plant saprotrophs and endophytes, while CK1 shows higher plant parasites/pathogens, leaf/wood saprotrophs, and ectomycorrhizal guilds. AV1–AV3 = intercropping at 0–10, 10–20, 20–30 cm; CK1–CK3 = monoculture at the same depths. Color scale −2 to +2.

Table S1 Bacterial taxonomic tallies by depth and cropping system: phylum to species levels detected from 16S V3-V4 ASVs.

| Treatment | Phylum | Class | Order | Family | Genus | Species |
| --- | --- | --- | --- | --- | --- | --- |
| AV1 | 44 | 134 | 283 | 428 | 707 | 809 |
| AV2 | 43 | 134 | 272 | 407 | 686 | 782 |
| AV3 | 41 | 130 | 270 | 399 | 613 | 677 |
| Total | 48 | 152 | 325 | 505 | 920 | 1117 |
| CK1 | 39 | 116 | 242 | 364 | 573 | 647 |
| CK2 | 33 | 96 | 185 | 268 | 375 | 409 |
| CK3 | 32 | 97 | 188 | 259 | 387 | 417 |
| Total | 41 | 122 | 261 | 397 | 666 | 771 |

Counts of unique taxa (cumulative per depth) detected in intercropped (Rubber/*Amomum villosum*) and monoculture (Rubber) soils. Sample codes: AV1–AV3 = intercropping at 0–10, 10–20, 20–30 cm; CK1–CK3 = monoculture at the same depths.

Table S2 Relative abundance (%) of soil bacterial phyla across depths and cropping systems

| Phylum | CK1 | CK2 | CK3 | AV1 | AV2 | AV3 |
| --- | --- | --- | --- | --- | --- | --- |
| p__Acidobacteriota | 30.83 | 34.01 | 26.01 | 11.84 | 18.81 | 19.33 |
| p__Chloroflexi | 17.83 | 27.89 | 33.23 | 4.77 | 14.17 | 15.15 |
| p__Planctomycetota | 16.50 | 7.19 | 9.47 | 32.44 | 10.39 | 15.49 |
| p__Proteobacteria | 12.17 | 9.71 | 9.50 | 16.56 | 16.37 | 13.45 |
| p__Actinobacteriota | 8.90 | 8.69 | 8.90 | 10.16 | 9.43 | 6.19 |
| p__Verrucomicrobiota | 3.63 | 2.69 | 2.51 | 5.81 | 5.22 | 4.66 |
| p__Firmicutes | 2.54 | 2.29 | 2.73 | 3.89 | 4.23 | 3.36 |
| p__Methylomirabilota | 1.26 | 1.71 | 2.04 | 1.61 | 4.76 | 5.85 |
| p__Gemmatimonadota | 1.31 | 0.90 | 0.66 | 2.41 | 6.04 | 5.34 |
| p__Myxococcota | 1.15 | 0.75 | 0.58 | 3.82 | 3.88 | 2.62 |
| p__Latescibacterota | 0.08 | 0.01 | 0.00 | 1.87 | 2.34 | 2.35 |
| p__Nitrospirota | 0.72 | 0.89 | 0.83 | 0.90 | 0.53 | 1.59 |
| p__GAL15 | 0.49 | 0.66 | 1.14 | 0.02 | 0.17 | 0.85 |
| p__Desulfobacterota | 0.30 | 0.27 | 0.24 | 0.46 | 0.45 | 0.34 |
| p__Patescibacteria | 0.24 | 0.06 | 0.09 | 0.89 | 0.26 | 0.48 |
| p__WPS-2 | 0.26 | 0.77 | 0.61 | 0.01 | 0.03 | 0.16 |
| p__Bacteroidota | 0.26 | 0.17 | 0.15 | 0.60 | 0.40 | 0.26 |
| p__Entotheonellaeota | 0.13 | 0.21 | 0.13 | 0.16 | 0.46 | 0.54 |
| p__NB1-j | 0.04 | 0.01 | 0.01 | 0.25 | 0.34 | 0.33 |
| p__MBNT15 | 0.01 | 0.00 | 0.00 | 0.14 | 0.18 | 0.16 |
| p__Cyanobacteria | 0.04 | 0.04 | 0.04 | 0.13 | 0.15 | 0.08 |
| p__Elusimicrobiota | 0.14 | 0.06 | 0.04 | 0.10 | 0.10 | 0.05 |
| p__RCP2-54 | 0.14 | 0.11 | 0.09 | 0.01 | 0.03 | 0.08 |
| p__Bdellovibrionota | 0.06 | 0.02 | 0.02 | 0.18 | 0.09 | 0.06 |
| p__Chlamydiae | 0.03 | 0.00 | 0.00 | 0.07 | 0.15 | 0.16 |
| p__Zixibacteria | 0.00 | 0.00 | 0.00 | 0.04 | 0.17 | 0.17 |
| p__Armatimonadota | 0.03 | 0.05 | 0.04 | 0.01 | 0.08 | 0.04 |
| p__FCPU426 | 0.02 | 0.01 | 0.02 | 0.05 | 0.03 | 0.03 |
| p__Dependentiae | 0.00 | 0.01 | 0.01 | 0.01 | 0.05 | 0.04 |
| p__SAR324_clade(Marine_group_B) | 0.01 | 0.00 | 0.00 | 0.04 | 0.02 | 0.01 |
| Others | 0.87 | 0.80 | 0.93 | 0.73 | 0.63 | 0.79 |

AV1–AV3 = intercropping at 0–10, 10–20, 20–30 cm; CK1–CK3 = monoculture at the same depths.

Table S3 Relative abundance of soil bacterial genera across depths and cropping systems

| Genus | CK1 | CK2 | CK3 | AV1 | AV2 | AV3 |
| --- | --- | --- | --- | --- | --- | --- |
| *g__HSB_OF53-F07* | 6.15 | 10.91 | 13.94 | 0.69 | 2.68 | 5.50 |
| *g__Acidothermus* | 6.04 | 6.61 | 7.07 | 2.10 | 2.84 | 1.55 |
| *g__ADurb.Bin063-1* | 0.90 | 0.57 | 0.45 | 1.79 | 1.93 | 1.29 |
| *g__Candidatus_Solibacter* | 1.72 | 1.13 | 0.86 | 0.89 | 1.02 | 0.87 |
| *g__BacC-u-018* | 0.84 | 1.52 | 3.13 | 0.01 | 0.13 | 0.51 |
| *g__Nitrospira* | 0.69 | 0.88 | 0.83 | 0.88 | 0.53 | 1.59 |
| *g__Candidatus_Udaeobacter* | 1.14 | 0.81 | 0.69 | 0.50 | 0.59 | 0.44 |
| *g__Haliangium* | 0.34 | 0.12 | 0.10 | 1.39 | 1.22 | 0.85 |
| *g__Candidatus_Xiphinematobacter* | 0.64 | 0.86 | 0.90 | 0.31 | 0.45 | 0.83 |
| *g__1921-2* | 1.01 | 1.14 | 1.58 | 0.01 | 0.07 | 0.11 |
| *g__MND1* | 0.12 | 0.00 | 0.01 | 1.51 | 1.25 | 0.76 |
| *g__Bryobacter* | 0.52 | 0.43 | 0.46 | 0.47 | 0.63 | 0.61 |
| *g__Ligilactobacillus* | 0.30 | 0.49 | 0.55 | 0.65 | 0.53 | 0.35 |
| *g__Bradyrhizobium* | 0.39 | 0.32 | 0.29 | 0.78 | 0.58 | 0.46 |
| *g__Lactobacillus* | 0.32 | 0.45 | 0.56 | 0.72 | 0.40 | 0.33 |
| *g__Acidibacter* | 0.56 | 0.38 | 0.26 | 0.52 | 0.52 | 0.40 |
| *g__FCPS473* | 0.58 | 0.92 | 0.68 | 0.04 | 0.12 | 0.25 |
| *g__Gemmata* | 0.18 | 0.01 | 0.12 | 1.55 | 0.25 | 0.24 |
| *g__1921-3* | 0.65 | 0.52 | 0.66 | 0.04 | 0.10 | 0.05 |
| *g__Akkermansia* | 0.22 | 0.20 | 0.28 | 0.51 | 0.30 | 0.30 |
| *g__Pirellula* | 0.06 | 0.00 | 0.00 | 1.12 | 0.25 | 0.36 |
| *g__Rhodoplanes* | 0.23 | 0.13 | 0.10 | 0.63 | 0.51 | 0.20 |
| *g__Pajaroellobacter* | 0.31 | 0.26 | 0.18 | 0.34 | 0.41 | 0.27 |
| *g__Clostridium_sensu_stricto_1* | 0.31 | 0.12 | 0.22 | 0.19 | 0.35 | 0.44 |
| *g__Aquisphaera* | 0.30 | 0.11 | 0.09 | 0.72 | 0.17 | 0.20 |
| *g__JG30a-KF-32* | 0.07 | 0.53 | 0.63 | 0.00 | 0.08 | 0.20 |
| *g__Anaeromyxobacter* | 0.22 | 0.04 | 0.04 | 0.47 | 0.50 | 0.22 |
| *g__Pedomicrobium* | 0.15 | 0.12 | 0.08 | 0.31 | 0.47 | 0.32 |
| *g__mle1-7* | 0.07 | 0.00 | 0.00 | 0.45 | 0.48 | 0.31 |
| *g__Mycobacterium* | 0.12 | 0.14 | 0.14 | 0.46 | 0.17 | 0.23 |
| Others | 74.86 | 70.24 | 65.10 | 79.97 | 80.50 | 79.96 |

AV1–AV3 = intercropping at 0–10, 10–20, 20–30 cm; CK1–CK3 = monoculture at the same depths.

Table S4 Fungal taxonomic tallies by depth and cropping system

| Treatment | Phylum | Class | Order | Family | Genus | Species |
| --- | --- | --- | --- | --- | --- | --- |
| AV1 | 7 | 23 | 62 | 124 | 250 | 382 |
| AV2 | 7 | 20 | 52 | 102 | 180 | 242 |
| AV3 | 7 | 20 | 53 | 101 | 171 | 227 |
| Total | 7 | 25 | 67 | 146 | 298 | 474 |
| CK1 | 7 | 26 | 53 | 101 | 165 | 230 |
| CK2 | 7 | 21 | 47 | 83 | 127 | 161 |
| CK3 | 6 | 17 | 39 | 73 | 108 | 135 |
| Total | 7 | 27 | 60 | 118 | 212 | 308 |

Counts of unique taxa (cumulative per depth) detected in intercropped and monoculture soils. Depths: 0–10, 10–20, 20–30 cm; n = 3 per depth × system.

Table S5 Relative abundance of the soil fungal phylum across depths and cropping systems

| Phylum | CK1 | CK2 | CK3 | AV1 | AV2 | AV3 |
| --- | --- | --- | --- | --- | --- | --- |
| Ascomycota | 72.32 | 75.79 | 62.07 | 68.98 | 79.13 | 83.25 |
| Basidiomycota | 20.82 | 6.33 | 29.05 | 24.97 | 15.54 | 9.33 |
| Fungi_unclassified | 4.72 | 13.35 | 4.97 | 3.39 | 2.96 | 3.38 |
| Zygomycota | 0.89 | 3.17 | 2.96 | 0.75 | 0.42 | 2.11 |
| Glomeromycota | 1.19 | 1.32 | 0.95 | 1.86 | 1.87 | 1.90 |
| Chytridiomycota | 0.06 | 0.04 | 0.00 | 0.05 | 0.07 | 0.03 |

AV1–AV3 = intercropping at 0–10, 10–20, 20–30 cm; CK1–CK3 = monoculture at the same depths.

Table S6 The relative abundance of soil fungal genera across depths and cropping systems

| Genus | CK1 | CK2 | CK3 | AV1 | AV2 | AV3 |
| --- | --- | --- | --- | --- | --- | --- |
| *Ascomycota_unclassified* | 64.91 | 61.21 | 50.70 | 64.91 | 61.21 | 50.70 |
| *Fungi_unclassified* | 4.72 | 13.35 | 4.97 | 4.72 | 13.35 | 4.97 |
| *Agaricales_unclassified* | 14.90 | 4.17 | 11.15 | 14.90 | 4.17 | 11.15 |
| *Cryptococcus* | 2.80 | 1.03 | 0.43 | 2.80 | 1.03 | 0.43 |
| *Agaricomycetes_unclassified* | 0.79 | 0.31 | 0.32 | 0.79 | 0.31 | 0.32 |
| *Psilocybe* | 0.02 | 0.02 | 13.46 | 0.02 | 0.02 | 13.46 |
| *Chaetomium* | 0.76 | 5.03 | 2.33 | 0.76 | 5.03 | 2.33 |
| *Hypocreales_unclassified* | 0.34 | 0.28 | 0.41 | 0.34 | 0.28 | 0.41 |
| *Sagenomella* | 0.46 | 3.93 | 2.15 | 0.46 | 3.93 | 2.15 |
| *Umbelopsis* | 0.48 | 2.72 | 2.65 | 0.48 | 2.72 | 2.65 |
| *Glomeromycetes_unclassified* | 0.84 | 1.05 | 0.88 | 0.84 | 1.05 | 0.88 |
| *Gymnopus* | 0.02 | 0.01 | 0.00 | 0.02 | 0.01 | 0.00 |
| *Nectriaceae_unclassified* | 0.08 | 0.03 | 0.12 | 0.08 | 0.03 | 0.12 |
| *Pezizomycotina_Incertae_sedis_unclassified* | 0.32 | 0.19 | 0.10 | 0.32 | 0.19 | 0.10 |
| *Basidiomycota_unclassified* | 0.14 | 0.15 | 0.13 | 0.14 | 0.15 | 0.13 |
| *Sordariomycetes_unclassified* | 1.01 | 0.37 | 0.60 | 1.01 | 0.37 | 0.60 |
| *Eurotiomycetes_unclassified* | 0.40 | 0.21 | 0.08 | 0.40 | 0.21 | 0.08 |
| *Ganoderma* | 0.05 | 0.22 | 2.82 | 0.05 | 0.22 | 2.82 |
| *Xylariales_unclassified* | 0.20 | 0.01 | 2.16 | 0.20 | 0.01 | 2.16 |
| *Ophiocordyceps* | 0.16 | 0.05 | 0.06 | 0.16 | 0.05 | 0.06 |
| *Mortierella* | 0.41 | 0.44 | 0.28 | 0.41 | 0.44 | 0.28 |
| *Calcarisporiella* | 0.41 | 1.17 | 0.83 | 0.41 | 1.17 | 0.83 |
| *Glomeraceae_unclassified* | 0.20 | 0.28 | 0.07 | 0.20 | 0.28 | 0.07 |
| *Acremonium* | 0.03 | 0.01 | 0.00 | 0.03 | 0.01 | 0.00 |
| *Talaromyces* | 0.24 | 0.75 | 0.57 | 0.24 | 0.75 | 0.57 |
| *Hypocrea* | 0.36 | 0.07 | 0.52 | 0.36 | 0.07 | 0.52 |
| *Exophiala* | 0.20 | 0.06 | 0.01 | 0.20 | 0.06 | 0.01 |
| *Pleosporales_unclassified* | 0.15 | 0.08 | 0.05 | 0.15 | 0.08 | 0.05 |
| *Lycoperdaceae_unclassified* | 0.01 | 0.00 | 0.01 | 0.01 | 0.00 | 0.01 |
| *Ceratobasidium* | 0.81 | 0.03 | 0.04 | 0.81 | 0.03 | 0.04 |
| Others | 3.79 | 2.77 | 2.12 | 3.79 | 2.77 | 2.12 |

AV1–AV3 = intercropping at 0–10, 10–20, 20–30 cm; CK1–CK3 = monoculture at the same depths.

Table S7 Effects of planting pattern and soil depth on the relative abundance of major bacterial and fungal phyla

| Phylum | Soil Depth | | Cropping Pattern | | Soil Depth × Cropping Pattern | |
| --- | --- | --- | --- | --- | --- | --- |
|  | F | P | F | P | F | P |
| Acidobacteriota | 4.623 | 0.032 | 92.954 | 0.000 | 6.626 | 0.012 |
| Chloroflexi | 16.540 | 0.000 | 61.435 | 0.000 | 0.682 | 0.524 |
| Planctomycetota | 9.751 | 0.003 | 7.651 | 0.017 | 1.624 | 0.238 |
| Proteobacteria | 2.689 | 0.108 | 24.108 | 0.000 | 0.680 | 0.525 |
| Actinobacteriota | 9.476 | 0.003 | 0.363 | 0.558 | 10.236 | 0.003 |
| Gemmatimonadota | 14.968 | 0.001 | 218.223 | 0.000 | 26.946 | 0.000 |
| Myxococcota | 13.107 | 0.001 | 303.421 | 0.000 | 4.495 | 0.035 |
| Nitrospirota | 7.003 | 0.010 | 2.905 | 0.114 | 7.666 | 0.007 |
| Glomeromycota | 0.254 | 0.780 | 13.683 | 0.003 | 0.380 | 0.692 |

Table S8 Effects of planting pattern and soil depth on the relative abundance of bacterial genera

| Genus | Soil Depth | | Cropping Pattern | | Soil Depth × Cropping Pattern | |
| --- | --- | --- | --- | --- | --- | --- |
|  | F | P | F | P | F | P |
| *HSB_OF53-F07* | 13.664 | 0.001 | 56.096 | 0.000 | 0.947 | 0.415 |
| *BacC-u-018* | 15.260 | 0.001 | 57.505 | 0.000 | 6.259 | 0.014 |
| *1921-2* | 4.357 | 0.038 | 153.799 | 0.000 | 2.391 | 0.134 |
| *Acidothermus* | 3.074 | 0.084 | 407.620 | 0.000 | 6.488 | 0.012 |
| *ADurb.Bin063-1* | 1.698 | 0.224 | 21.718 | 0.001 | 0.547 | 0.592 |
| *CandidatusXiphinematobacter* | 8.227 | 0.006 | 11.524 | 0.005 | 1.773 | 0.212 |
| *Candidatus Solibacter* | 9.508 | 0.003 | 14.411 | 0.003 | 10.355 | 0.002 |
| *Gemmata* | 5.272 | 0.023 | 7.837 | 0.016 | 3.667 | 0.057 |
| *Pirellula* | 37.180 | 0.000 | 130.397 | 0.000 | 27.805 | 0.000 |
| *Haliangium* | 10.172 | 0.003 | 188.355 | 0.000 | 2.425 | 0.130 |
| *Nitrospira* | 7.293 | 0.008 | 3.016 | 0.108 | 7.745 | 0.007 |
| *MND1* | 3.738 | 0.055 | 75.613 | 0.000 | 2.244 | 0.149 |

Table S9 Effects of planting patterns and soil depth on the relative abundance of fungal genera

| Genus | Soil Depth | | Cropping Pattern | | Soil Depth * Cropping Pattern | |
| --- | --- | --- | --- | --- | --- | --- |
|  | F | P | F | P | F | P |
| *Cryptococcus* | 5.996 | 0.016 | 27.408 | 0.000 | 0.436 | 0.656 |
| *Chaetomium* | 1.604 | 0.241 | 7.448 | 0.018 | 1.591 | 0.244 |
| *Sagenomella* | 20.190 | 0.000 | 70.071 | 0.000 | 18.471 | 0.000 |
| *Umbelopsis* | 4.659 | 0.032 | 24.158 | 0.000 | 3.452 | 0.065 |
| *Gymnopus* | 1.765 | 0.213 | 1.737 | 0.212 | 1.743 | 0.216 |
| *Ophiocordyceps* | 5.756 | 0.018 | 41.552 | 0.000 | 3.599 | 0.060 |
| *Exophiala* | 4.645 | 0.032 | 3.758 | 0.076 | 2.291 | 0.144 |
| *Cladophialophora* | 1.294 | 0.310 | 2.727 | 0.125 | 0.233 | 0.796 |

Table S10 FUNGuild annotations of differential fungal taxa identified by LEfSe

| Phylum | Genus | Trophic mode | Guild |
| --- | --- | --- | --- |
| Glomeromycota | *Glomeraceae_unclassified* | Symbiotroph | Arbuscular mycorrhizal |
| Glomeromycota | *Glomeromycetes_unclassified* | Symbiotroph | Arbuscular mycorrhizal |
| Ascomycota | *Exophiala* | Saprotroph | Plant saprotroph |
| Basidiomycota | *Cryptococcus* | Saprotroph | Undefined saprotroph |
| Ascomycota | *Ophiocordyceps* | Symbiotroph | Endophyte |
| Ascomycota | *Sagenomella* | Saprotroph | Undefined saprotroph |
| Ascomycota | *Chaetomium* | Saprotroph | Plant saprotroph |
| Zygomycota | *Umbelopsis* | Saprotroph | Undefined saprotroph |
| Ascomycota | *Calcarisporiella* | Saprotroph | Undefined saprotroph |
| Basidiomycota | *Gymnopus* | Saprotroph | wood saprotroph |

Table S11 Good’s coverage values per treatment (mean ± SD, n=3)

| Treatments | Bacterial | Fungal |
| --- | --- | --- |
| AV1 | 1.00±0.00 | 1.00±0.00 |
| AV2 | 0.99±0.00 | 1.00±0.00 |
| AV3 | 1.00±0.01 | 1.00±0.00 |
| CK1 | 1.00±0.00 | 1.00±0.00 |
| CK2 | 1.00±0.00 | 1.00±0.00 |
| CK3 | 1.00±0.00 | 1.00±0.00 |

AV1, AV2, AV3 = soil sampled at 0–10, 10–20, 20–30 cm soil depth, respectively, for rubber-*A. villosum* intercropping system; CK1, CK2, CK3 = soil sampled at 0–10, 10–20, 20–30 cm soil depth, respectively, for monoculture rubber.
